# Effect of financial incentives on hospital-cardiologist integration and cardiac test location

**DOI:** 10.1111/jels.12359

**Published:** 2023-07-04

**Authors:** Andy Ye Yuan, Bernard Black, Timea Viragh, David J. Magid, Qian Luo, Ali Moghtaderi

**Affiliations:** 1 Pritzker School of Law, Northwestern University, Chicago, Illinois, USA; 2 Pritzker School of Law and Kellogg School of Management, Northwestern University, Chicago, Illinois, USA; 3 School of Education and Social Policy, Northwestern University, Evanston, Illinois, USA; 4 Division of Cardiology, University of Colorado, Anschutz Medical Campus, Aurora, Colorado, USA; 5 Milken Institute School of Public Health, George Washington University, Washington, DC, USA

**Keywords:** cardiac test, cardiologists’ integration, financial incentive, health policy

## Abstract

Starting around 2006, the Centers for Medicare and Medicaid Services (CMS) progressively reduced Medicare Fee-for-Service (M-FFS) payments for the principal noninvasive cardiac tests, when performed in a cardiologist office (Office), yet kept payments flat to increasing for the same tests, performed in the hospital-based outpatient (HBO) setting. This produced a growing gap between HBO and Office payments for the same tests, and thus an incentive for hospitals to acquire cardiology practices in order to move cardiac tests to the HBO location and capture the HBO/Office payment differential. We use difference-in-differences analysis, in which we compare national M-FFS trends in cardiac test location to those for a control group of several large, integrated Medicare Advantage (M-Adv) health systems over 2005–2015, which were not affected by these reimbursement changes, and provide evidence that these reimbursement changes led to a large shift in testing from Office to HBO. This shift was concurrent with a sharp rise in hospital-cardiologist integration. The rise in integration and the proportion of testing in HBO varied greatly across states. Independent practice remains viable in very large states, but is endangered in many states, and is all but extinct in a growing number of states.

## INTRODUCTION

**FIGURE 1:**
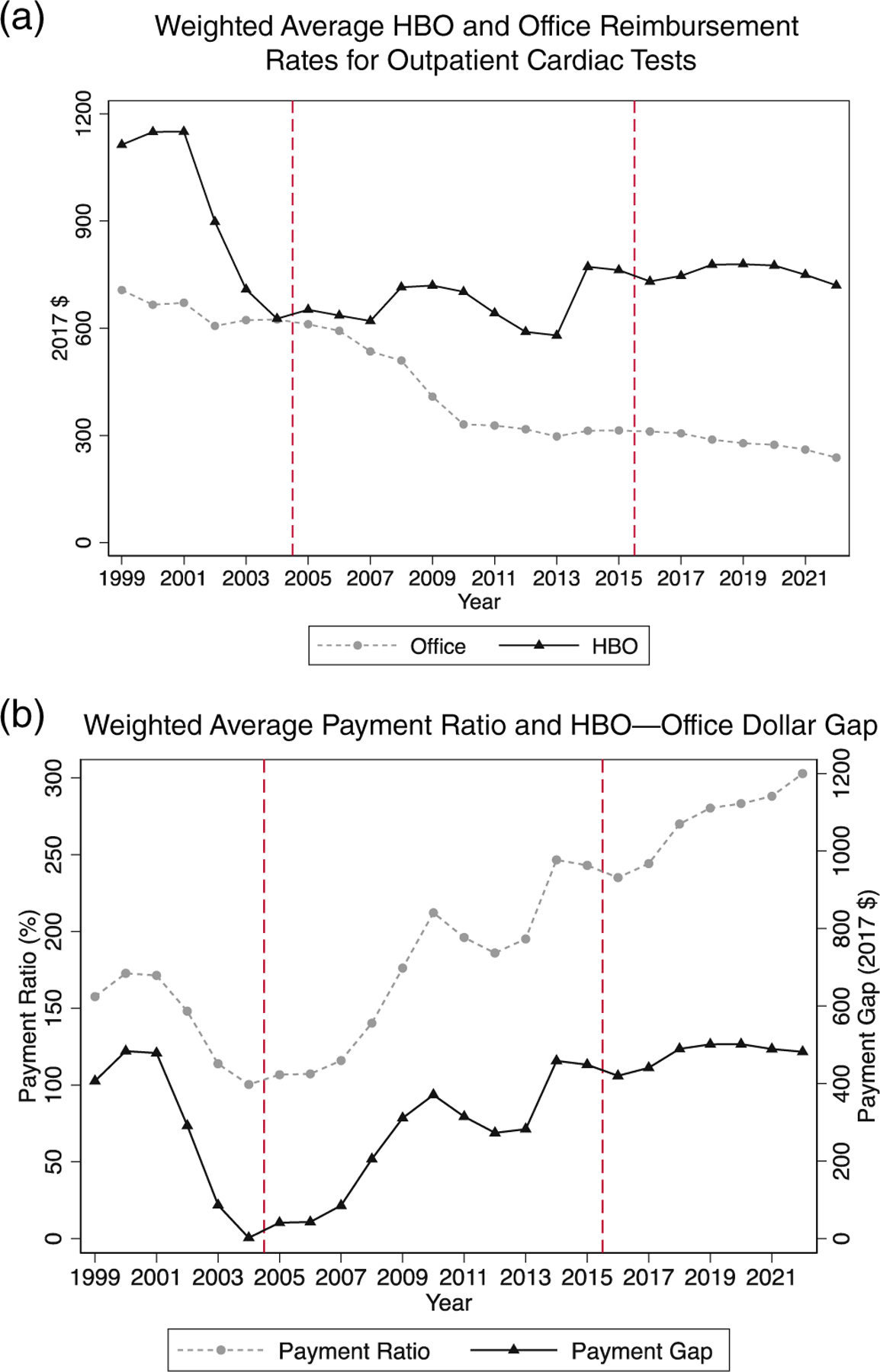
Office and hospital-based outpatient (HBO) payments for cardiac testing, 1999–2021. (a) Weighted average Medicare Fee-for-Service payment rates for indicated cardiac tests performed in the HBO or Office location over 1999–2021, based on the Physician Fee Schedule obtained from the Centers for Medicare and Medicaid Services website, in 2017 dollars. (b) Weighted average HBO/Office Payment Ratio (left-hand *y*-axis) and weighted average dollar gap between HBO and Office payment levels (right-hand *y*-axis). Vertical lines between 2004–2005 and 2015–2016 indicate the start and end of our sample period. See Appendix S1 for specific test codes we use and how we compute weighted average payment.

In the mid-2000s, many people believed that cardiac imaging tests, conducted in cardiologist offices (Office location), were highly profitable, and that provided incentives for overuse of these tests (Ferrari et al., 2014; Medicare Payment Advisory Commission, 2007, 2010). In response, the Centers for Medicare and Medicaid Services (CMS) progressively reduced Medicare Fee-for-Service (M-FFS) payment levels for Office-based tests, with the sharpest cuts over 2007–2010 (Figure 1a). Weighted average Office payment for the principal cardiac imaging tests fell (all amounts in this paper are in 2017 dollars), from $624 in 2004 to $408 in 2009, $331 in 2010, and $260 in 2021.^1^ In contrast, over the same period, M-FFS payment rates for these tests performed in hospital-based outpatient departments (HBO location) setting remained flat or increased; the weighted average payment was $627 in 2004 and $750 in 2021.

Medicare reimbursement is the sum of a physician professional fee and reimbursement for what CMS calls the “technical component” of the test—the estimated cost of testing equipment, support staff, and ancillary services (also called a facility fee). The physician receives the same amount for conducting the test, regardless of location. However, the facility fee can depend on location. Over our sample period, CMS progressively reduced Office facility fees, while maintaining or increasing HBO facility fees.

The result was a dramatic rise in both the dollar difference between HBO and Office payments (“Payment Gap”) and the ratio of average HBO to Office payments, converted to a percentage (Payment Ratio), which rose from approximately 100% over 2004–2006 to 288% in 2021. Yet, these are the same tests, often performed by the same people, using similar equipment. Plausible extra facility costs in the HBO setting are far less than the difference in facility fees.

An anecdote: At Northwestern Memorial Hospital during this period, Northwestern-employed cardiologist faculty conducted Office testing on the 23rd floor of a building across the street from the hospital, and HBO testing on the 8th floor of the same building. Same building, same people, same equipment, minor variations in room layout, and ventilation. When the HBO-Office payment differential became substantial, Northwestern Memorial quickly realized that it was more profitable to conduct cardiac testing on the eighth floor and proceeded to do so.

We use Medicare claims data to study how differential M-FFS payments for the same outpatient imaging tests, in Office versus HBO, affected test location and vertical integration of cardiologists with hospitals. We hypothesize that the growing Payment Gap led to more tests being performed in HBO setting, and to hospitals employing more cardiologists, as the means through which hospitals could move tests to the HBO location and capture the extra revenue from doing so. The Payment Gap allowed hospitals to offer higher compensation to employed cardiologists, relative to the amounts the cardiologists could earn in independent practice. We developed a simple conceptual model illustrating the trade-off between financial benefits and loss of autonomy when physicians decide whether to become employed or remain in independent practices (Berenson, 2017; Post et al., 2018).

To estimate the causal effect of the M-FFS financial incentives on cardiac test location, we use a difference-in-difference (DiD) research design, in which we compare trends in test location for M-FFS to trends in a control group of three large Medicare Advantage (“M-Adv”) plans run by three major, integrated health maintenance organizations (HMOs): Kaiser Permanente Colorado, Kaiser Permanente Northwest (Oregon and southwest Washington), and Group Health (based in Washington). M-Adv plans are an alternative to traditional M-FFS, which provide care for similar patients, but are paid by Medicare on a capitation rather than a fee-for-service basis. The capitation payments do not depend on the number or location of the cardiac tests performed by the M-Adv sites. The M-Adv group is an appropriate control group to examine the causal effects of M-FFS financial incentives. Both M-FFS and M-Adv should be affected by national trends in cardiac health and healthcare, including changes in the relative efficiency of the Office and HBO locations. However, the M-FFS payment changes will affect only M-FFS patients. Our M-Adv plans rely on employed physicians and receive capitated payments to treat patients seeking cardiac care. Therefore, changes in M-FFS payment are unlikely to affect the cardiac care practice in M-Adv plans.

We examine the predictive effect of financial incentives on test location using both continuous measures (the Payment Ratio and the Payment Gap) and a binary measure (a dummy variable that we turn on either in 2008 or in 2010 [dropping 2008–2009 as a transition period]), with consistent results.

We report evidence that the rising payment differential induced a large shift in cardiac test location from Office to HBO for M-FFS patients. The national proportion of M-FFS tests conducted in the HBO location (the “HBO Proportion”) roughly doubled from 20% in 2007 to over 40% by 2015, yet was flat or slightly declining in M-Adv. One unit increase in Payment Ratio predicts about 16-percentage-point increase in the HBO Proportion.

We also provide evidence on hospital-cardiologist integration and the principal means by which hospitals were able to move tests to HBO and capture the payment differential. The timing of the rise in integration, beginning soon after the Payment Ratio begins to rise, and the close association among the Payment Ratio, the HBO Proportion, and integration support a causal relationship, with the rising payment differential driving both integration and test location.

We explore interstate heterogeneity in the shift to integrated cardiology practice, and report evidence that the shift is more limited in states in the highest quartile for testing rates. We hypothesize that in these states, standalone cardiology practices are more likely to be able to maintain sufficient test volumes to sustain independent practice.

Our study fits within a literature that examines the impacts of changes in M-FFS reimbursement policy on healthcare delivery, including the impact of higher HBO reimbursement on hospital-physician integration (Dranove & Ody, 2019; Ferrari et al., 2014; Forlines, 2018; Masoudi et al., 2019; Nikpay et al., 2018; Song et al., 2015). Ferrari et al. (2014) provided evidence on the Payment Gap. Nikpay et al. (2018) document the trend toward hospital-physician integration. Masoudi et al. (2019) described the parallel trends toward both more HBO testing and hospital-cardiologist integration. Post et al. (2018) find a modest positive relationship between the HBO-Office payment differential and hospital-physician integration, which was mostly concentrated among primary care physicians and a few specialties, including cardiologists. We find a much larger association between payment differentials and hospital-cardiologist integration, likely reflecting our using a better measure of integration.

Dranove and Ody (2019) and Song et al. (2015) are the closest to this project. Both papers model incentives for integration as due to a one-time increase in the payment differential in 2010, using an interrupted time series design, with no control group. In contrast, we document the gradual change in M-FFS reimbursement policy for cardiac imaging tests, which began in 2007 and continued after 2010, and have an M-Adv control group. This use of a DiD research design. This paper is also the first to examine heterogeneous trends across states in cardiologist integration and the movement of cardiac tests to HBO.

Our paper is also related to research on the price and quality effects of vertical integration, which include reduced competition between providers (Capps et al., 2017), increased self-referral (Baker et al., 2016), higher prices (Capps et al., 2018; Ciliberto & Dranove, 2006; Dafny et al., 2019), higher medical spending (Baker et al., 2014; Neprash et al., 2015, 2017; Scheffler et al., 2018), and potentially lower quality of care (Carlin et al., 2015; Eliason et al., 2020; Scott et al., 2017). Our paper also contributes to the broader literature on how financial incentives affect healthcare delivery (e.g., Alexander, 2020; Clemens & Gottlieb, 2014; Yurukoglu et al., 2017), and research on between M-FFS and M-Adv in cardiac testing rates (Matlock et al., 2013) and rates for invasive cardiac procedures (e.g., Avraham & Schanzenbach, 2015; Chandra & Staiger, 2007, 2020; Koch et al., 2018).

Our paper provides evidence that differential payment rates, together with the Stark laws, which prohibit hospitals from directly paying physicians to move testing to HBO, can have unintended effects on the industrial organization of healthcare by spurring to vertical integration, which then affects healthcare cost and quality. It is also related to proposals for site-neutral Medicare payment rates, for those tests and procedures that can be safely performed in either HBO or Office (e.g., BlueCross BlueShield Association, 2023; MedPAC, 2018). Reducing HBO reimbursement to the generally lower Office levels has been estimated to reduce Medicare spending by around $100 billion annually (Committee for a Responsible Federal Budget, 2023). For often-elective procedures, such as the cardiac tests we study, there could be further savings if lower reimbursement reduces testing rates, against the background that M-FFS testing rates are roughly double M-Adv rates, suggesting substantial overtesting in M-FFS (Masoudi et al., 2019). Vertical integration can also lead to higher prices paid by commercial insurers.

The rest of the paper proceeds as follows. The second section presents a conceptual framework that illustrates the tradeoff between financial profits and loss of autonomy when physicians determine whether to change test location from Offices to HBO settings. The third section describes relevant data and summary statistics. The fourth section presents the empirical framework. The fifth section describes the main results for the effects of financial incentives on test location. The sixth section discusses hospital-cardiologist integration. The seventh section presents the heterogeneous effects analysis. The eighth section discusses our results.

## CONCEPTUAL FRAMEWORK

We develop in this section a simple model of hospitals’ decisions to seek to acquire cardiology practices, and cardiologists’ decisions to remain in independent practice, versus to integrate with a hospital or health system (become hospital-employed). The model formalizes cardiologists’ tradeoff between financial gains from integration and loss of independence.^2^ We do not model the incentives of cardiologists in smaller practices to integrate horizontally into larger practice groups, as an alternative to vertical integration with a hospital or health system.

A starting point for the model is federal anti-kickback rules, which prohibit hospitals from paying cardiologists for moving imaging tests to the HBO location.^3^ M-FFS reimbursement for physician services does not depend on test location, and hospitals cannot offer side payments to physicians for conducting tests in the HBO location. However, the anti-kickback rules do not affect the overall salary arrangements between hospitals and employed physicians. A hospital *cannot* pay employed cardiologists more for conducting tests in the HBO location, but *can* simply instruct the employed cardiologists to do so when feasible. Integration is thus the primary means through which hospitals can move tests from Office to HBO.

Let $r_{H}$ and $r_{O}$ represent the Medicare reimbursement for a cardiac test conducted in HBO and Office location, respectively. We consider only one type of test for simplicity. The variable cost of conducting a test is c, and is assumed to be the same in both locations.^4^ We ignore the one-time fixed cost to hospitals and physicians of the acquisition, including moving test location, physicians learning a different billing system, etc. The hospital profit from conducting q tests is $\pi_{H}\left(q\right)=\left(r_{H}-c\right)q$. To persuade cardiologists to become hospital-employed, the hospital needs to share a fraction δ of the additional revenue with the cardiologists, where δ must be large enough so that the cardiologists earn higher utility from being hospital-employed than from being self-employed. The hospital net gain after these payments is $\left(r_{H}-r_{o}\right)\times q\times \left(1-\delta \right)$.

The compensation paid by Medicare to cardiologists for conducting q tests in Office location is $\pi_{o}\left(q\right)=\left(r_{o}-c\right)q$. Let k be the positive utility associated with physicians’ independence from hospitals, which will depend on compensation, workload, work environment, and other factors, and will vary across physicians (Berenson, 2017). Cardiologists will integrate to hospitals if the extra compensation from doing so exceeds the utility of remaining independent:

$$\left(r_{H}-r_{o}\right)\times q\times \delta >k.$$

As the payment differential $\left(r_{H}-r_{o}\right)$ increases, so does the economic rent that can be captured by the hospital through integration, and thus the hospital’s incentive and ability to strike a deal with cardiologists to persuade them to integrate, in which the payment differential is shared between the hospital and employed cardiologists.

## BACKGROUND

### Datasets and covariates

For M-FFS patients, we rely on two core datasets for measuring integration and test location. The first is the 5% M-FFS random sample, covering Medicare part A (hospital services) and part B (both physician services and HBO services), over 1999–2019, which overlaps with our M-Adv dataset over 2005– 2015. There are three principal M-FFS datasets: Inpatient claims are included in the MedPAR file; HBO claims are in the Outpatient file, and physician claims are in the Carrier file. We study M-FFS recipients age 65+ in the 50 states plus the District of Columbia. A Medicare “denominator file” indicates by month which beneficiaries are M-FFS or M-Adv; we exclude M-Adv recipients. As beneficiaries leave the sample (principally through death), the sample is “refreshed” with new beneficiaries, about 70% of whom are age 65 when added to our sample. While this dataset includes only 5% of M-FFS patients, it should include virtually all physicians who see these patients.

The second dataset is a database of providers that we constructed in separate work, which includes provider specialty and identifies, by year, which cardiologists are hospital-employed (Luo et al., 2023; Moghtaderi et al., 2023). See details below.

We obtain M-Adv data from three major managed care groups for 2005 through the third quarter of 2015: Kaiser Permanente Colorado (KP CO); Kaiser Permanente Northwest (KP NW); and Group Health (GH), for 2005–2015. These data cover around 125,000 M-Adv beneficiaries in three states (Colorado, Washington, and Oregon) in 2005; the number of patients rises to 188,000 in 2015.^5^ We use the M-Adv sites as a “control group” for the M-FFS environment. The M-Adv sites are a good control group for test location because they are paid based on a per-patient “capitated” payment for all care provided to their enrollees. They are not reimbursed on a per-test basis, and are not affected by changes in M-FFS reimbursement rates. Thus, (i) the M-Adv sites have incentives to perform cardiac testing in the more efficient location for each patient, as between HBO and Office; and (ii) changes in M-FFS payment levels should not affect M-Adv testing rates or locations. However, we are unable to use cardiologists working at the M-Adv sites as a control group to estimate the effects of financial incentives on integration decisions because the M-Adv sites are vertically integrated systems, and cardiologists working at these sites are integrated through the sample period.

In regressions, we include the following patient-level covariates: dummy variables for each of the 17 measures included in the Charlson comorbidity index,^6^ age in years, gender (reference group is “male”), White, Black, Hispanic, or other race (reference group is White). We measure comorbidities using a rolling four-quarter lookback period (the quarter in which a cardiac test is carried out, through the date of the test, plus the preceding four calendar quarters).

### Measures

We summarize our measures here; see Appendix S1 for additional details.

#### Noninvasive cardiac tests studied

We study the most common outpatient noninvasive cardiac tests (NCTs), including both stress and non-stress tests. We do not study inpatient testing. We study the following noninvasive tests: transthoracic echocardiography (TTE) and transesophageal echocardiography (TEE) (together, echocardiography, or *echo*); stress electrocardiography (*stress ECG*); stress echocardiography (*stress echo*); and single photon emission computed tomography (*SPECT*).^7^ All tests can be performed in either HBO or Office location.

#### Measuring M-FFS payment levels and testing rates

To obtain data on payment levels, we rely primarily on the Medicare Physician Fee Schedule (PFS), available from the CMS website for 2000–2021.^8^ As a check on our calculations, we use two alternate sources (i) payment levels for stress echo, stress ECG and resting echo, reported annually by the American Society of Echocardiography (ASE) for 2004–2018; and (ii) actual patient and CMS payments to providers, extracted from M-FFS claims files for 1999–2015. We confirm consistency across all three sources. See calculation details and consistency checks in Appendices A and D. We convert all amounts to 2017 dollars.

#### Measuring testing rates

We measure testing rates as tests per 1000 patient-years.^9^ We generate indicator variables for each distinct test (=1 on the test day, 0 otherwise), compute rates for each, and sum the rates to obtain a combined “All Cardiac Test” rate. For the three M-Adv sites, we weigh each site equally.^10^

#### Identifying which cardiac tests are office-based

In the M-FFS data, each NCT is performed by a physician, who bills for the “professional” component of the test in the Carrier file (modifier “26” will appear in the claim). M-FFS pays separately for a “technical component” which covers providing the testing equipment and performing the test. This payment will appear in the Carrier file for Office tests (modifier TC will appear in the claim). For tests performed in the HBO location, the hospital will bill for the technical component in the Outpatient file. For the M-Adv data, each site coded each claim as inpatient, HBO, or Office.

#### Identifying cardiologists and measuring integration

We identify cardiologists who saw M-FFS patients using a database of cardiologists that we prepared for a separate project (Moghtaderi et al., 2023). Overall, we identify 41,475 cardiologists who billed Medicare FFS for services over 1999–2019 in our Medicare dataset. Appendix B provides more details on how we identify cardiologists. Because we use Medicare data to identify which cardiologists are integrated, we can study only tests performed on the elderly, by cardiologists who see Medicare patients.^11^

A core need is to measure accurately which physicians are integrated. There are four main measures used in the literature, but none is satisfactory. One method assesses whether the tax identification number (TIN) that physicians use to bill belong to a hospital (Capps et al., 2018; Ho et al., 2020). This measure generates many false negatives due to the lack of a comprehensive source for hospital TINs. Ho et al. (2020), in a case study of Texas, find that their TIN-based measure identifies only 71% of integrated physicians. A second measure uses the place of service that physicians use for billing (Neprash et al., 2015, 2017; Post et al., 2018). This approach treats a cardiologist as integrated if the cardiologist predominantly performs outpatient NCTs in the HBO location. However, this method can generate many false positives. For example, cardiologists in smaller, nonintegrated practices often do not purchase their own imaging equipment and instead perform outpatient NCTs in the HBO setting. The third method uses a database prepared by IQVIA (formerly SK&A) to measure vertical integration (e.g., Nikpay et al., 2018). This method can also generate many false positives especially when only current data are used to determine integration status. A principal matching variable that IQVIA uses to link across data sources is the provider’s business name (often an informal name, not the legal business name). When practice ownership changes, the TIN often changes to a hospital-associated TIN; however, the practice will often maintain its former business name. Also, if only the latest version of IQVIA data is used to determine integration status (often the case due to the high cost of obtaining annual data from IQVIA), many practices that became integrated in later years will be misclassified as integrated in earlier years.^12^ The fourth source is the Compendium of U.S. Health Systems prepared by the Agency for Healthcare Research and Quality (AHRQ). This source provides information about the physician practice affiliation with hospital and health systems. This source is available only for 2016 and 2018, and generates many false positives because it effectively measures affiliation rather than integration.^13^

In response to the limitations of prior measures of hospital-physician integration, we develop in separate work a new measure of vertical integration, in which we draw on a wide variety of sources to identify hospital-associated TINs (Luo et al., 2023). Here, we apply that measure to cardiology.

We classify cardiologists as integrated in year t if they bill for at least 75% of evaluation and management (E&M) services in that year using a hospital-associated TIN; nonintegrated if this percentage is less than 25%; and mixed if this percentage is at least 25% but less than 75%. We treat cardiologists as *switching* from nonintegrated to integrated if this percentage rises from <25% in year t to at least 75% in year t+1 or year t+2; and as switching from integrated to nonintegrated if this percentage falls from at least 75% in year t to less than 25% in year t+1 or t+2.

### Summary statistics

**FIGURE 2:**
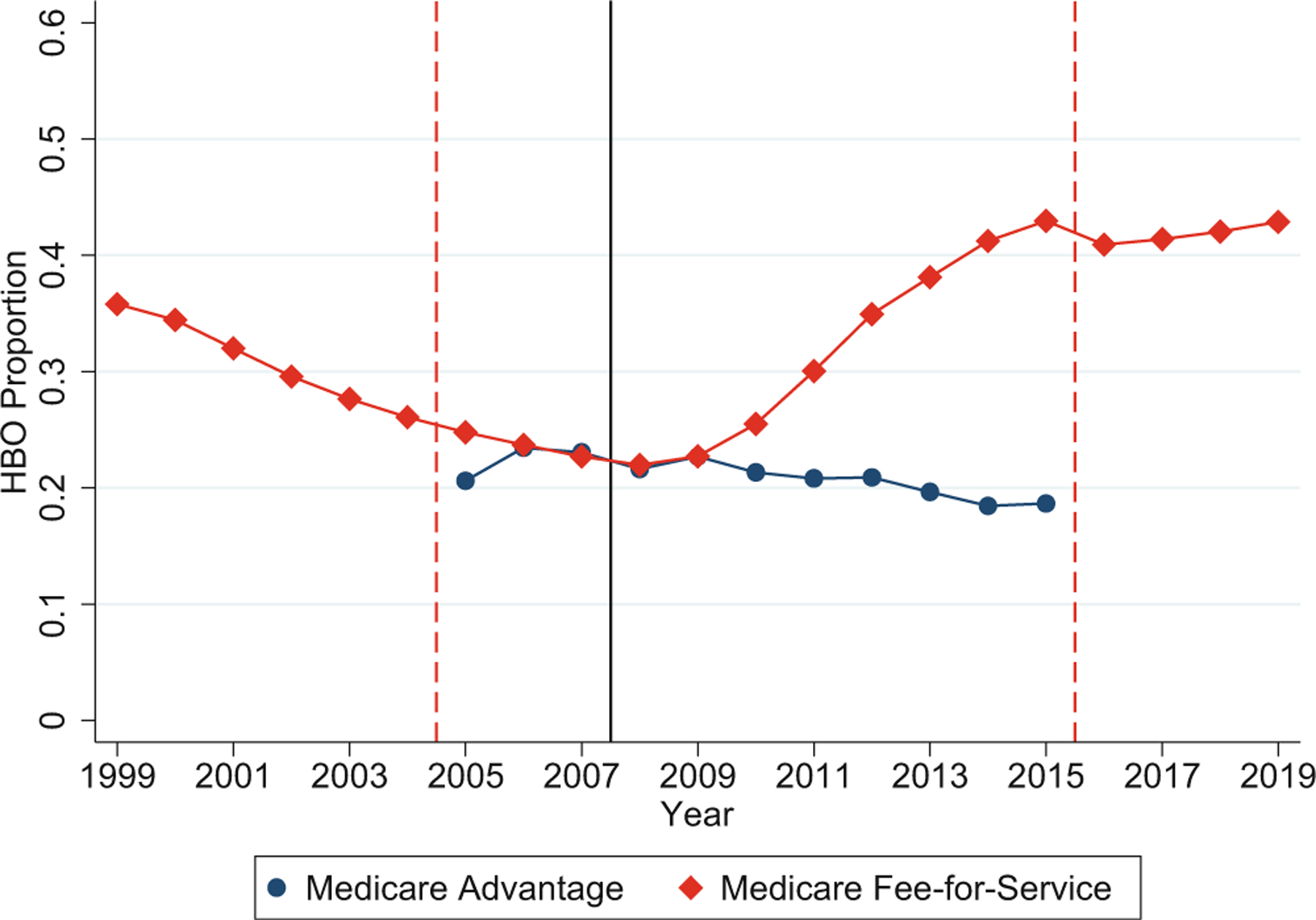
Hospital-based outpatient (HBO) Proportion for Medicare Fee-for-Service (M-FFS) versus Medicare Advantage (M-Adv). HBO Proportion for M-FFS over 1999–2019 and M-Adv over 2005–2015 is shown. Each data point represents the proportion of cardiac tests performed in HBO settings. Dashed vertical lines between 2004–2005 and 2015–2016 indicate the start and end of our sample period. Solid vertical line between 2007 and 2008 indicates start of the treatment period.

Table 1 reports summary statistics for the key variables for M-FFS and M-Adv patients. For M-FFS, we report data separately for all states and for the three M-Adv states. For each variable, the table also reports the standardized mean difference between the M-FFS and M-Adv subsamples. In Panel A, the national M-FFS sample is substantially larger than the M-Adv sample, but M-Adv sample size is still ample to provide a control group for M-FFS patients. We discuss first the differences between the national M-FFS sample and the M-Adv sample. The HBO Proportion is higher, on average, for M-FFS, but as we show below, this is driven by a higher proportion in the later sample years when the Payment Ratio is high. The HBO Proportion was very similar for the two groups during years when the Payment Ratio was close to 1 (see Figure 2). Thus, the M-Adv sites provide a control group that experienced similar test location decisions when payment for HBO versus Office testing was similar.

Panel B of Table 1 compares demographics and comorbidities for the M-FFS and M-Adv patients. There are some differences between the two samples, although none appear so large as to compromise the use of M-Adv as a control group, as long as one controls for patient demographics in regressions. M-FFS patients are 0.7 years older on average and have somewhat more comorbidities than M-Adv patients; are more likely to be female (56% vs. 50%), more likely to be Black (7% vs. 3%) but less likely to be Hispanic (2% vs. 4%) or another race (4% vs. 9%). The standardized differences range from −0.21 (for other race) to +0.10 (for age). Due to the large sample, all differences are statistically significant at the 1% level.

Panel C of Table 1 provides summary statistics for annual cardiac testing rates at M-FFS versus M-Adv sites. National M-FFS testing rates are much higher than M-Adv rates (215 total tests per 1000 patients for M-FFS sites vs. 101 for M-Adv sites). M-FFS testing rates are higher for all tests except stress ECG. This comparison is consistent with prior evidence that, compared with M-FFS, M-Adv plans have lower utilization of cardiac care services, including testing and screening (Curto et al., 2019) and invasive procedures (Matlock et al., 2013).

There are some important differences between the national and three-state M-FFS samples. The three-state sample has lower comorbidities, a higher proportion of women and Whites, and fewer Blacks and Hispanics. Overall M-FFS cardiac testing rates in the three states are well above M-Adv rates, but well below national average rates.

## EMPIRICAL STRATEGY: DiD MODELS

We begin with our analysis with the following specification:

(1) $$y_{ist}=\alpha_{0}+\alpha_{1}{\text{MFFS}}_{it}+\alpha_{2}{\text{Payratio}}_{t}+\alpha_{3}{\text{MFFS}}_{it}\times {\text{Payratio}}_{t}+X_{ist}\gamma +\delta_{s}+\beta_{t}+\in_{ist}.$$

This can be seen as a “continuous-DiD” specification (Atanasov & Black, 2016), that uses a continuous Payment Ratio but assumes that this ratio is exogenously determined. Here i indexes observations (patient-years with a cardiac test), s indexes state, t is calendar year. The outcome variable $y_{ist}$ is the test location (=1 if HBO, =0 if Office). If a patient has multiple tests in 1 year, we treat each test as a separate observation. We study both a combined measure of “Any Cardiac Test” and each test type separately. ${\text{MFFS}}_{it}$ is a dummy variable that equals 1 if test i was conducted a M-FFS site. ${\text{Payratio}}_{t}$ is a weighted average of the HBO/Office payment amount in year t, weighted by the fraction of tests of each type conducted in M-FFS, averaged over 2005–2007 (considered as the pre-treatment period). In robustness checks, we use the weighted average payment gap as an alternative measure of hospital financial incentives to move testing to HBO and allow the weights on each test type to vary based on actual test proportions in each year. $X_{it}$ is a vector of patient characteristics: age dummies, gender, race (White, Black, other, and unknown), ethnicity (Hispanic or non-Hispanic), and comorbidities (dummy variables for each of the 17 elements of the Charlson comorbidity index). The $\delta_{s}$ are state fixed effects (FEs), which control for time-invariant, state-specific unobserved characteristics that might affect test location choice. The $\beta_{t}$ are year FEs, which we include in selected specifications, but otherwise omit because Payment Ratio, is set annually by CMS on a nationwide basis, so year FEs will absorb Payment Ratio. Standard errors are clustered at state level.

The parameter $\alpha_{1}$ measures the difference in likelihood of a test being performed in HBO setting between M-FFS and M-Adv in the three M-Adv states, when the Payment Ratio equals 1. The parameter $\alpha_{2}$ captures the predictive effect of Payment Ratio for the likelihood of a cardiac test being performed in the HBO setting for M-Adv patients. We do not expect the Payment Ratio to have a causal effect on M-Adv test location, because it does not affect the amounts paid to M-Adv sites for treating Medicare patients.

The key parameter of interest is $\alpha_{3}$, which measures the change in likelihood of a test being performed in HBO setting for M-FFS patients in response to change in Payment Ratio. A positive coefficient on $\alpha_{3}$ is expected if a higher Medicare reimbursement for HBO testing indeed lead to more tests being performed in HBO settings. Integration is the principal means by which hospitals can move tests to the HBO location, and will not happen instantly, so we expect test location to respond to Payment Ratio with a lag. In regressions below, we allow for lags of up to 3 years. A core identifying assumption for our national analysis is that, with state FEs and controls for patient demographics and comorbidities, the M-Adv systems provide a reasonable counterfactual for national M-FFS testing rates, even though we have M-Adv data from only three states.

To interpret $\alpha_{3}$ as a causal estimate, we need to assume parallel trends: In absence of any differences in incentives (Payment Ratio = 1), the likelihood of performing a cardiac test in the HBO location would have followed similar trends for M-FFS and M-Adv patients. This assumption is untestable. In a DiD setting with a binary treatment, one can assess the plausibility of this assumption by seeing whether trends are parallel for the treatment and control groups during the pre-treatment period. However, under Equation (1), we cannot test for parallel trends, even during the period when the Payment Ratio was relatively stable, because $payratio_{t}$ is continuous. To assess the evidence for pre-treatment parallel trends, we adopt a DiD specification:

(2) $$y_{ist}=\beta_{0}+\beta_{1}{\text{MFFS}}_{it}+\beta_{2}\text{Post\_2008}+\beta_{3}{\text{MFFS}}_{it}\times \text{Post\_2008}+X_{ist}\gamma +\delta_{s}+\beta_{t}+\in_{ist}.$$

Compared with Equation (1), we replace the continuous treatment variable, ${\text{Payratio}}_{t}$, with a binary variable, Post_2008, indicating whether a cardiac test was conducted during or after 2008. This assumes that the financial incentives for moving tests to HBO were small during the initial sample years of 2005– 2007, but large thereafter. This assumption reflects the actual Office and HBO payment levels by year, shown in Figure 1. Below, we also use an alternate specification in which we drop 2008 and 2009 as transition years and replace Post_2008 with Post_2010.

The key parameter of interest in this specification is $\beta_{3}$, which captures the average effect of the financial incentive on the likelihood of a cardiac test being performed at HBO settings among M-FFS patients. We use two event-study models to assess trends over time and whether trends appear parallel in the pre-treatment period. The first extends Equation (1) and uses Payment Ratio as a predictor; the second extends Equation (2) using is similar but uses Post_2008 as the predictor. The models are:

(3a) $$y_{ist}=\beta_{0}+\beta_{1}{\text{MFFS}}_{it}+\beta_{2}{\text{Payratio}}_{t}+\sum_{k=200}^{2015}\left(\lambda_{k}{\text{MFFS}}_{it}\times {\text{Payratio\_Year}}_{k}\right)+X_{ist}\gamma +\delta_{s}+\beta_{t}+\in_{ist}.$$

(3b) $$y_{ist}=\beta_{0}+\beta_{1}{\text{MFFS}}_{it}+\beta_{2}\text{Post\_2008}+\sum_{k=-3}^{6}\left(\lambda_{k}{\text{MFFS}}_{it}\times {\text{Post\_2008}}_{k}\right)+X_{ist}\gamma +\delta_{s}+\beta_{t}+\in_{ist}.$$

Consider Equation (3a). ${\text{Payratio\_Year}}_{k}$ is a dummy variable that equals Payment Ratio in the year which is k year(s) before or after 2008, and 0 otherwise; k takes values from −3 (2005) to +6 (2014). The omitted year is 2007. Parameters of interests are the $\lambda_{k}$; we set $\lambda_{-1}=0$. Parallel pretreatment trends imply that the $\lambda_{k}$ during the pre-treatment periods should be small magnitude and statistically insignificant. The $\lambda_{k}$ estimates during the treatment period map out the estimated treatment effect over time; we hypothesize that this effect will increase due to both the generally increasing Payment Ratio, and the lag between when the rising Payment Ratio creates incentives to move tests to HBO, and when hospital carry out vertical integration, to make it feasible to move test location. Equation (3b) is similar except we substitute ${\text{Post\_2008}}_{k}$ for ${\text{Payratio\_Year}}_{k}$.^14^

## MAIN RESULTS

### HBO versus office payments over time

Figure 1a shows weighted average M-FFS payment levels for the four cardiac tests we study, separately for Office and HBO locations, over 1999–2021.^15^ Figure 1b reports the Payment Ratio over this period. Vertical lines indicate the start and end of the sample period, for which we have both M-FFS and M-Adv data (2005–2015). The Payment Ratio was well above 1 during 1999–2002 (prior to our core sample period) because CMS was reimbursing, by mistake, two add-on codes for SPECT at a similar rate to the main test in HBO settings.^16^ When CMS reduced the SPECT add-on payments, Office and HBO payments were similar over 2003–2006. After that, HBO payment grows steadily relative to Office, driven largely by declining Office payment for the technical components of the cardiac tests, and partially by higher absolute HBO payments, relative to 2007, during 2008–2010 and again beginning in 2014. Despite all the controversy around these Payment differentials, the gap between Office and HBO setting grew in 2013 and remained stable through 2021. In Figure 1b, we report the weighted average HBO/Office Payment Ratio using the left-hand *y*-axis, and the dollar difference using the right-hand *y*-axis. The Payment Ratio is close to 100% for 2003–2006, but rises steadily after that. The Payment Gap in per-test reimbursement is only $43 in 2006 but grows substantially after that.^17^

The large gap between HBO and Office payments has attracted substantial criticism, including calls to return to the rough payment parity that prevailed between 2004 and 2006. However, as Figure 1 shows, the Payment Ratio has continued to rise through 2021 including substantial jumps in 2014 and 2018; the 2021 level was 288%. The Payment Gap rose over 2006–2014 and has been fairly flat since then. The 2021 gap was $489.

### Time trends in test location

Figure 2 shows the annual proportions of cardiac tests performed in the HBO location for M-FFS over 1999–2019 and for M-Adv over the period for which we have M-Adv data, from 2005 to 2015. We expect test location to respond to the Payment Ratio with a lag. Dashed vertical lines show the start and end of the sample period; a solid vertical line shows the assumed start of the treatment period (2008). During 1999–2004 (prior to our main sample period, where we have only M-FFS data), the HBO Proportion of cardiac tests conducted in M-FFS sites fell steadily, loosely corresponding to the falling payment gap. This decline continues through 2008.

The HBO Proportion is similar for M-FFS and M-Adv sites over 2005–2009 and is in the 20%–25% range. Since M-Adv sites have incentives to conduct NCTs in the lower-cost location, this suggests that the Office location is preferred for most patients. Figure 2 also confirms that when the Payment Ratio is close to 1, the HBO Proportion is similar between M-Adv and M-FFS. This is consistent with the M-Adv sites being a good control group for the M-FFS sites.

Figure 2 shows that the HBO Proportion rises for M-FFS relative to M-Adv beginning in 2010. This is consistent with a rising Payment Ratio driving integration with a roughly 2-year lag, with integration then leading to (being the mechanism for) moving tests to HBO. The HBO proportions of cardiac tests performed at M-FFS sites increased sharply from 22% in 2009 to around 44% in 2015, and roughly maintained that level through 2019. In contrast, there is, if anything, a slightly decreasing trend in the HBO proportions of cardiac tests conducted at M-Adv sites. The HBO Proportion was 21% in 2005, rose to 23.4% in 2006, and then gradually declined to 19% in 2015.^18^

### DiD analysis: Financial incentives and test location

We turn in Table 2 to a continuous-DiD design, following Equation (1). Panel A shows full-sample results (50 states plus DC). Panel B shows the results for the three states where our M-Adv sites are located. Both panels use similar regression specifications. The national analysis uses analytical standard errors clustered on state and report standard errors. For the three-state analysis, we address the small number of clusters by using the wild-cluster bootstrap (Cameron & Miller, 2015; MacKinnon & Webb, 2020) and cluster standard errors at the state × treatment status level (six clusters), and report bootstrapped *p*-values. Simulation results from MacKinnon and Webb (2020) suggest that with six clusters, these *p*-values will be slightly conservative.

In Panel A, Column (1) presents results from a simple specification that includes an M-FFS dummy and patient demographics and comorbidities but does not include either the Payment Ratio or state FEs. The interpretation is that, on average over our sample period, M-FFS patients are about 10% more likely to receive a test in HBO setting than M-Adv patients (not statistically significant). In Column (2), we add state FE. Recall that we have M-Adv data only for three states. For the other 48 states, the average HBO Proportion over our sample period will be absorbed by the state FE. Column (2) thus shows that on average, over our sample period, M-FFS patients in these three states were 25.0% more likely to receive a test in HBO setting relative to M-Adv patients in the same state $\left(p<\;0.001\right)$.

Column (3) includes two additional main regressors, Payment Ratio and Payment Ratio interacted with M-FFS dummy. The point estimate for M-FFS dummy is now −0.03, and statistically insignificant. The noninteracted Payment Ratio also takes a small and insignificant negative coefficient. The interpretation is that in the three M-Adv states, the HBO Proportion is similar when the Payment Ratio is close to 1, and that the Payment Ratio does not predict the HBO Proportion for the M-Adv sites—as we expect and require if the M-Adv sites are to provide a valid control group. The variable of principal interest is the interaction term. The coefficient for the interaction term is 0.16 and highly statistically significant. Thus, a one-point increase in the Payment Ratio predicts a 16-percentage-point increase in the HBO Proportion. In Column (4), we add year FEs, which absorb Payment Ratio; the coefficient on the interaction term changes only slightly.

Columns (5)–(7) show results for 1, 2, and 3 lags of Payment Ratio and the interaction term. Using lags is appropriate if hospitals require time to acquire cardiology practices and then move NCT location. The coefficient on the interaction term rises with each additional lag, reaching 0.222 with three lags. The negative coefficient on noninteracted Payment Ratio is consistent with the gradual fall in the HBO Proportion for M-Adv states, over our sample period (Figure 2).

The three-state results in Panel B are consistent with the national results, but with larger magnitudes. In Column (3), the coefficient for the interaction term, MFFS × Payment Ratio, implies that a one-point increase in Payment Ratio predicts a 33% increase in the HBO Proportion. Also, the estimated coefficient for MFFS dummy is −0.3 and highly statistically significant. This suggests that, compared with M-Adv patients, M-FFS patients are 30% less likely to receive cardiac tests at HBO settings when Payment Ratio equals 1.

### Dollar impacts of NCT payment gap

In this section, we estimate the extra combined cost to CMS and patients due to NCTs moving from Office to HBO. The baseline for the estimate is the combined cost for the same tests if the HBO Proportion in M-FFS had been the same as in M-Adv, using the actual annual M-Adv proportion, HBO and Office reimbursement rates, and testing rates.^19^ For each NCT type, we compute the number of extra NCTs performed in the HBO location, multiply by the Payment Gap for that type of test, then sum across test types.

**FIGURE 3:**
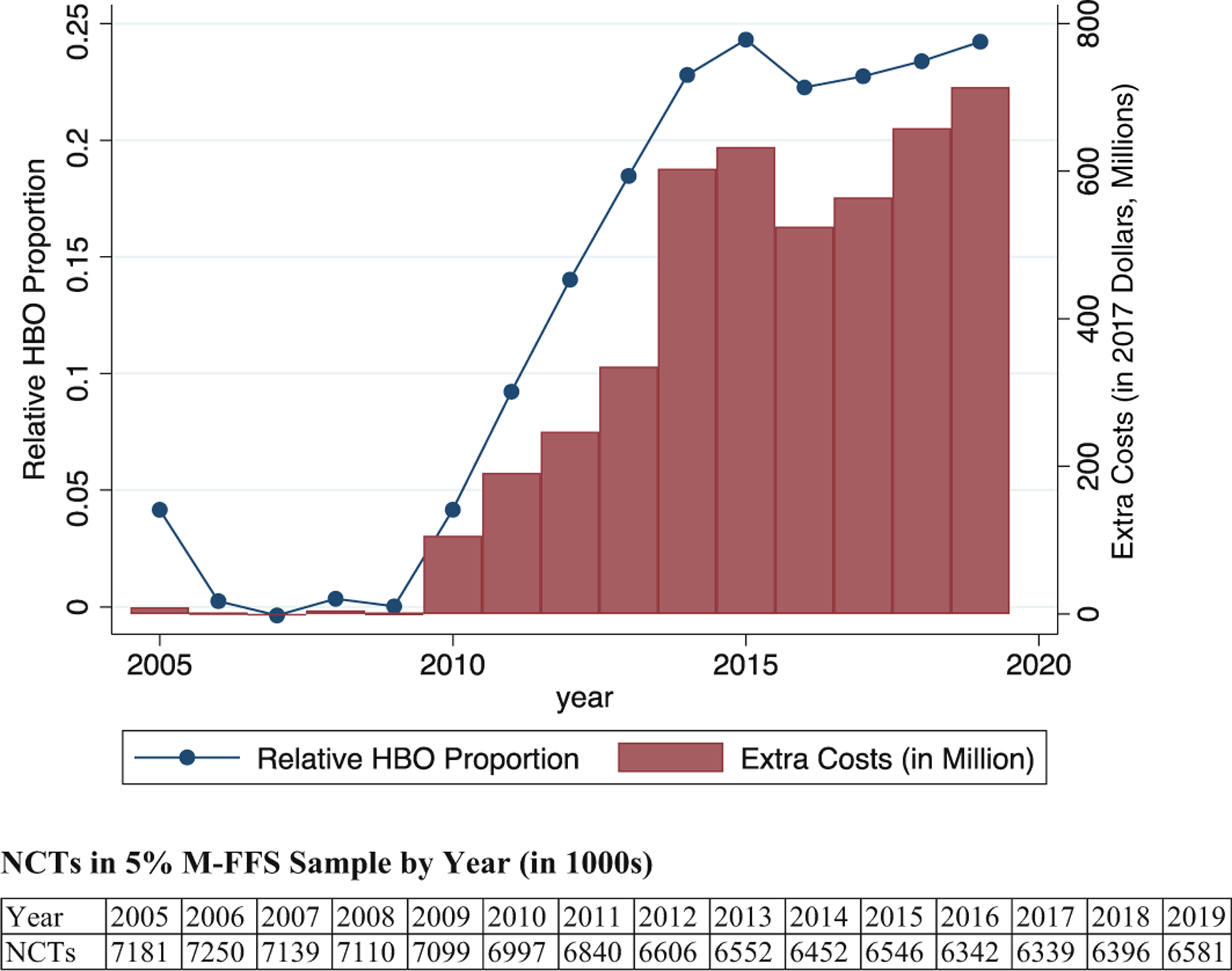
Dollar impacts of the Medicare reimbursement policy. Hospital-based outpatient (HBO) Proportion in Medicare Fee-for-Service (M-FFS) relative to Medicare Advantage (left-hand *y*-axis) and extra cost of noninvasive cardiac tests (NCTs) relative to cost for same tests if relative HBO Proportion were zero (right-hand *y*-axis) are shown. Small table below the figure shows the number of NCTs in the 5% M-FFS sample by year. Number of observed tests is multiplied by 20 to convert from the 5% sample to a national estimate.

Figure 3 shows the estimated extra combined cost. The figure shows the difference between the HBO Proportion in M-FFS and the proportion in M-Adv (left-hand *y*-axis); and the extra combined cost due to higher HBO payments (right-hand *y*-axis). A small table underneath the figure shows the number of NCTs by year in the 5% M-FFS sample. Figure 3 indicates that, if these same tests had been performed, with the same payment amounts but no test migration, total NCT cost would have been lower by around $600 million annually over 2014–2019.

CMS reduced Office reimbursement levels because it believed that Office testing was highly profitable for cardiologists and therefore overused. If Office and HBO payment levels had both been reduced, maintaining pay parity between them, some tests that moved from Office to HBO might not have taken place. To the extent that testing rates would have fallen, Figure 3 underestimates the hypothetical savings from reducing NCT payment levels in both locations.

### Replacing payment ratio with a binary variable

We next replace continuous Payment Ratio with a dummy variable for the period starting in 2008, in which the Payment Ratio was substantially above one. Table 3, Panel A shows results for a national sample (including 50 states and DC). Panel B shows the results for a subsample of three states where we have both M-FFS and M-Adv claims. Both panels use Equation (2).

Subpanel A.1 of Table 3 presents regression results for the interaction between an M-FFS dummy and a Post-2008 dummy. We estimate five regression specifications in which we progressively add controls for patient demographics, comorbidities, state FEs, and year FEs. The preferred specification is either Column (4), where we include all controls except year FE, or Column (5), where we include year FEs, which absorb Post-2008 dummy. The M-FFS dummy becomes significant in Column (4) where it indicates that, prior to 2008, for the three states where we have M-Adv claims, M-FFS patients are 16.6% more likely than M-Adv patients to receive a cardiac test in the HBO setting. The small, insignificant coefficient on Post-2008 dummy indicates that there were no significant changes in test location for M-Adv patients after versus before 2008; the negative sign is consistent with the gradual decline in the HBO Proportion for M-Adv patients shown in Figure 2.

**FIGURE 4:**
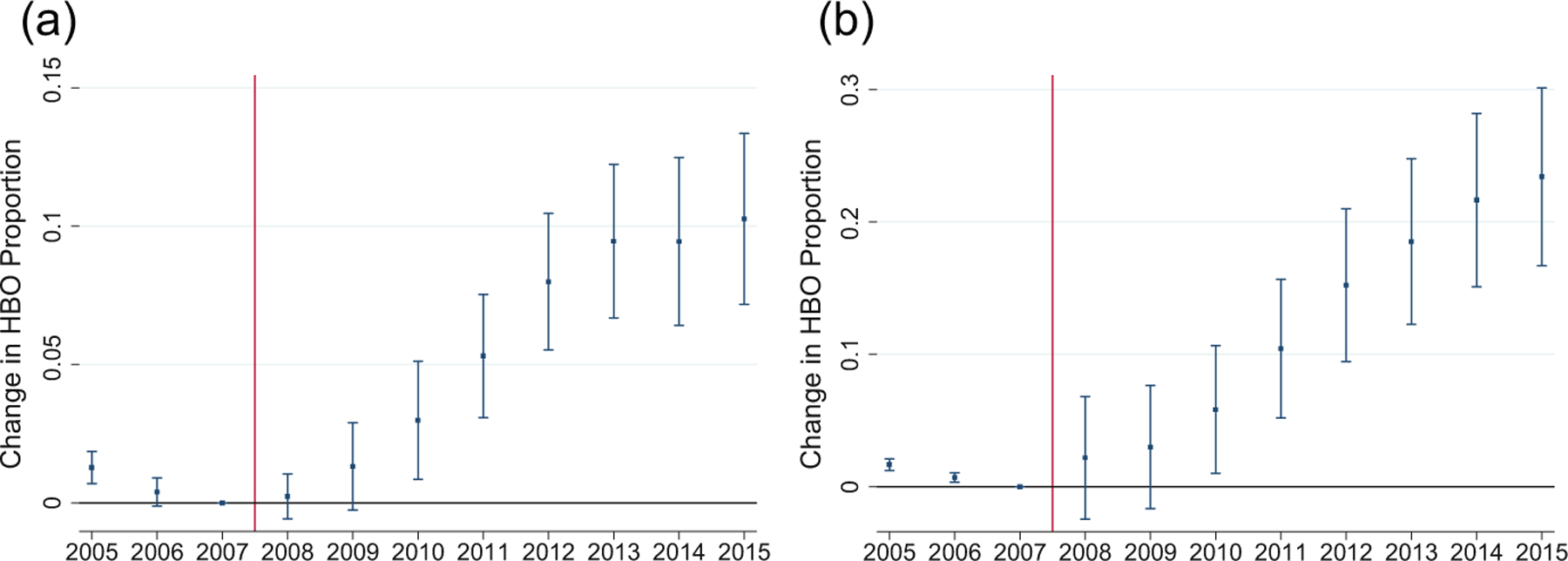
Event study graph for the effects of Payment Ratio on test location. The sample includes Medicare Fee-for-Service claims from all 50 states plus DC and Medicare Advantage claims from three states. The outcome variable is whether the location of a cardiac test is hospital-based outpatient (HBO) or office. The model includes state fixed effects, patient demographics, and dummy variables for comorbidities. Standard errors are clustered at the state level. Year 2007 is omitted, with the coefficient pegged to zero. (a) The annual event study coefficients for Payment Ratio, using Equation (3a). (b) Coefficients for Post-2008 dummy, using Equation (3b). The 95% confidence intervals are shown as vertical lines. Long vertical line between 2007 and 2008 separates pre-treatment and treatment periods.

The coefficient on the interaction term is consistent across specifications and is 0.115 (highly significant) in Column (4). Thus, on average over 2008–2015, there was a roughly 12% increase in the likelihood that M-FFS patients who received NCTs would receive them in the HBO setting. In Subpanel A.2, the corresponding estimate is higher at 0.155 after excluding 2008–2009, as expected given the gradual emergence of the treatment effect shown in Figure 4.

The three-state results in Panel B are consistent with the national results, with larger magnitudes. In Column (4) of Subpanel B.1, the estimated coefficient for the interaction term is 0.228, which implies that the HBO Proportion increased by 22.8% in the post-2008 period. The coefficient increased to 0.306 after excluding 2008 and 2009.

### Event study approach and parallel pre-treatment trends

We also conduct an event study analysis, using Equations (3a) and (3b), and report results in Figure 4. Figure 4a reports results using Payment Ratio and Figure 4b reports results using the binary, Post-2008 predictor. In both figures, the pre-2008 trends are reasonably parallel, which supports the parallel trends assumption underlying our DiD analysis. For both figures, the coefficient on the interaction term turns positive in 2008 and rises steadily after that.^20^ The steady rise in the annual coefficients likely reflects a combination of two effects: A lag between the appearance of a meaningful payment gap and an effect on test location; and the increase in this gap over time.

### Robustness check: Alternative measures of financial incentives

We re-estimated Equation (1) using the national sample and three alternative measures of financial incentives: ln(Payment Ratio), Payment Gap, and ln(Payment Gap). Appendix Tables E.1–E.3 report results for each measure, in a format similar to Tables 2 and 3. Across all measures, higher reimbursement for NCTs in the HBO setting predicts a higher HBO Proportion.

## MECHANISM: VERTICAL INTEGRATION OF CARDIOLOGISTS WITH HOSPITALS

In this section, we explore the mechanism underlying the large shift in NCT from Office to HBO settings among M-FFS patients that corresponds to the rising Payment Ratio. As discussed above, to move tests and gain M-FFS revenue, the principal way that hospitals can move NCTs to the HBO location is by acquiring cardiology practices; federal law bars them from paying cardiologists more for moving NCTs to the HBO location. We therefore predict that a higher Payment Ratio will predict hospital-cardiologist integration.

**FIGURE 5:**
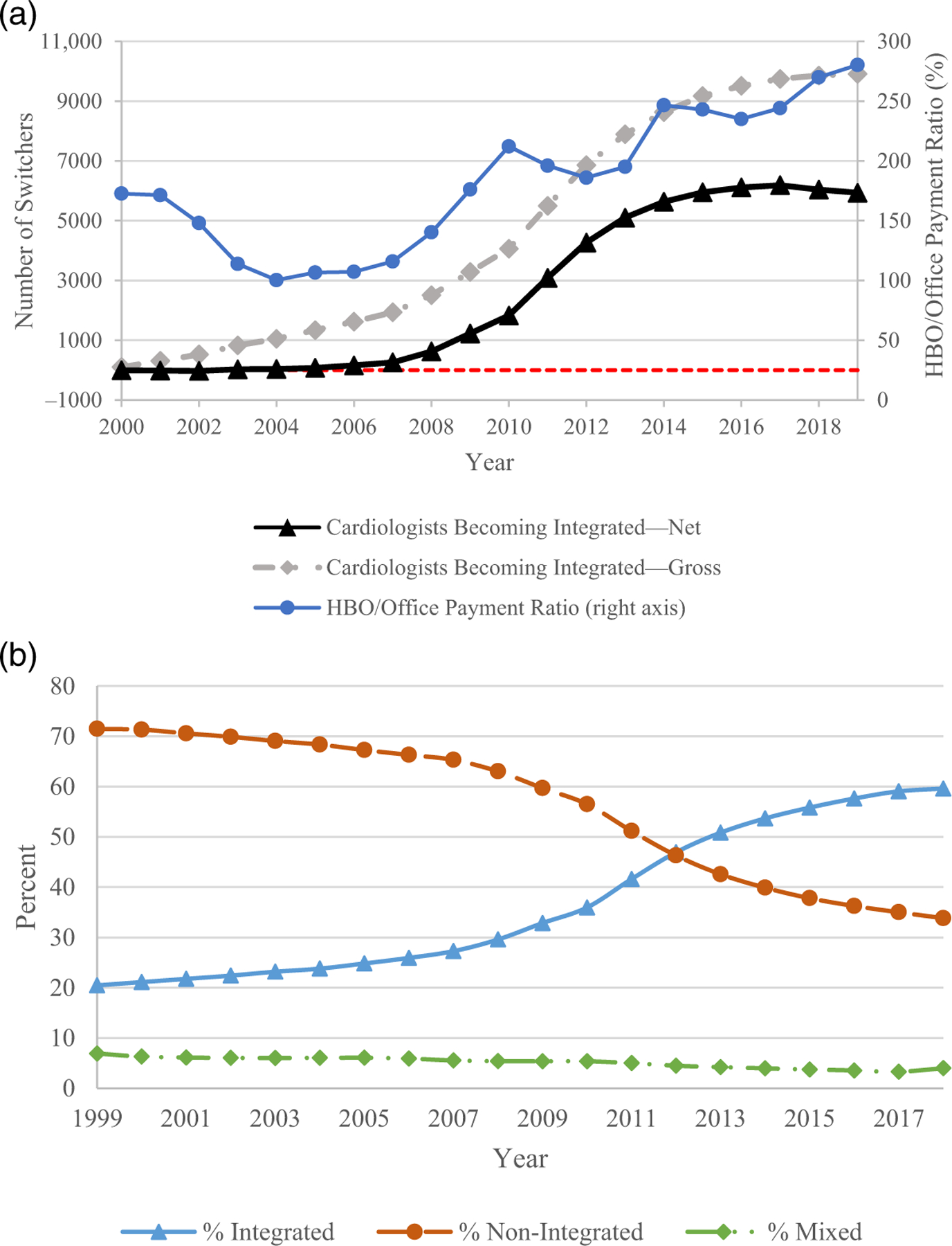
Cardiologist integration and hospital-based outpatient (HBO)/office Payment Ratio. (a) Gross and net switching. Figure shows: (i) the average HBO/Office Payment Ratio for noninvasive cardiac tests (NCTs); and (ii) gross and net numbers of cardiologists who switch from being nonintegrated to integrated, over 2002–2018. We treat cardiologists as switching from nonintegrated to integrated status in year $t+1\;\left(t+2\right)$ if the percentage of their bills for NCTs and evaluation and management (E&M) services using a hospital-associated tax identification number (TIN) rises from <25% in year t to >75% in year $t+1\;\left(t+2\right)$; and as switching from integrated to nonintegrated in year $t+1\;\left(t+2\right)$ if this percentage falls from >75% in year t to <25% in year $t+1\;\left(t+2\right)$. Dashed line shows 0 level. (b) Percent of integrated cardiologists. Figure shows annual percentage of cardiologists who are integrated (bill for at least 75% of their services using a hospital-associated TIN), nonintegrated (bill for less than 25% of their services using a hospital-associated TIN), or mixed (bill for 25%–75% of services using a hospital-associated TIN).

Figure 5a shows: (1) the average HBO/Office Payment Ratio for NCTs; and (2) the gross and net numbers of cardiologists who switch from being non-integrated to integrated, over 2000–2019. The net number of switchers is slightly negative over 2000–2002. Out of 41,475 cardiologists in the sample, 9910 cardiologists switch from being nonintegrated to integrated over 2000–2019, while 3974 move from being integrated to nonintegrated. Thus, there is a net increase of 5936 in the number of integrated cardiologists. The number of net switchers begins to rise in 2008 and accelerates in 2009, lagging slightly the rise in the Payment Ratio, but consistent in timing with the increase in the HBO Proportion. Figure 5b shows the annual proportion of cardiologists who are integrated, non-integrated, or mixed, over 1999–2018. The proportion of integrated cardiologists rose slowly prior to 2007, began to increase more rapidly after 2007, and flattened after 2015. Over the full period, the proportion of active integrated cardiologists tripled, from 20% to 60%.

**FIGURE 6:**
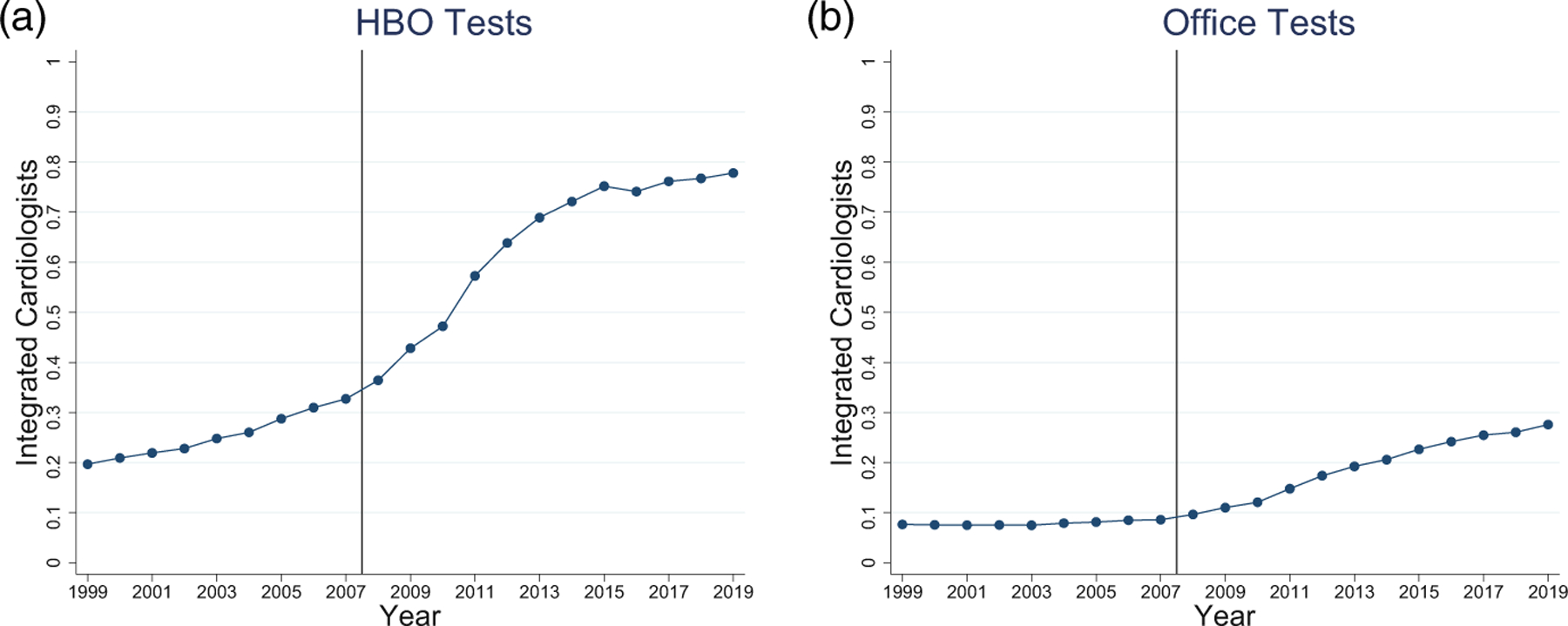
Proportions of hospital-based outpatient (HBO) and office tests performed by integrated versus non-integrated cardiologists in HBO and office settings. Sample is Medicare Fee-for-Service claims over 1999 and 2019. Solid vertical line between 2007 and 2008 indicates start of the treatment period. (a) The proportions of HBO tests performed by integrated cardiologists. (b) The proportions of office tests performed by integrated cardiologists.

Figure 6 shows the percentage of cardiac tests performed by integrated and nonintegrated cardiologists in each setting. Figure 6a presents the percentage of HBO tests performed by integrated cardiologists. This percentage increases from 30% in 2005 to around 75% in 2015, with the trend toward HBO tests being performed by integrated cardiologists accelerating beginning in 2009. This percentage then rises only gradually through 2019, consistent with the flattening in the HBO Proportion in Figure 2. In Figure 6b, the percentage of Office tests performed by integrated physicians also rises, but more slowly, from 9% in 2005 to almost 30% in 2019. Apparently, even for integrated cardiologists, other factors can sometimes lead to performing an NCT in the lower-paying Office location. In Appendix S1, we find similar patterns across all four types of tests.

In addition, we examined the association between financial incentives and the likelihood that an NCT will be performed in HBO by *an integrated cardiologist* by estimating the following linear probability model, applied to the M-FFS sample:

(4) $$y_{ist}=\alpha_{0}+\alpha_{1}{\text{Incentive}}_{t}+X_{ist}\gamma +\delta_{s}+\in_{ist}.$$

The outcome variable is whether a cardiac test is performed by an integrated cardiologist. Similar to Equations (1) and (2), $X_{ist}$ is a vector of the observed patient characteristics and $\delta_{s}$ is a vector of state FEs. We use as measures of financial incentive, Payment Ratio, Post-2008 dummy, and Post-2010 dummy. The main coefficient of interests is $\alpha_{1}$.

Table 4 presents regression results. The format of Table 4 is similar to Table 3, where we progressively add patient demographics, patient comorbidities, and state FE to the regression. Panel A uses Payment Ratio as the predictor variable; Panel B uses a Post-2008 dummy, and Panel C drops 2008 and 2009 and uses a Post-2010 dummy as the predictor variable. The estimated coefficients are insensitive to specification. In Panel B, Column (4), the likelihood that an NCT will be performed by an integrated cardiologist increased by 24 percentage points after 2008 (29 percentage points if we exclude 2008 and 2009).

**FIGURE 7:**
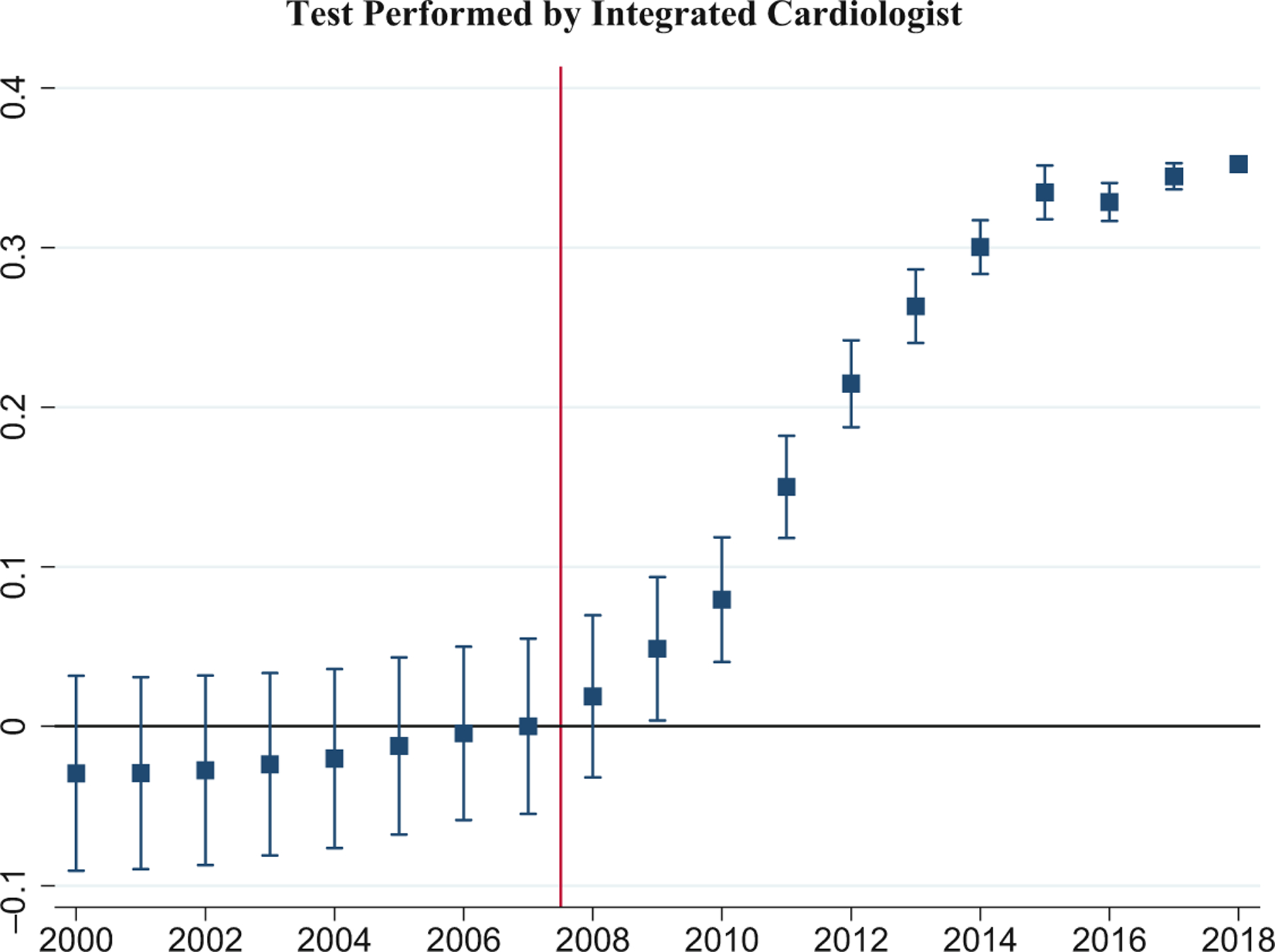
Financial incentives and tests performed by integrated cardiologists. The sample is Medicare Fee-for-Service claims over 1999–2019. The outcome is a dummy variable for whether a cardiac test is performed by an integrated cardiologist. The regression model includes state and year fixed effects (FEs), and patient demographics and comorbidities. The graph plots the coefficients on the year FEs, with 95% confidence intervals. The coefficient for 1999 is dropped because the regression model controls for patients’ comorbidities which are unobserved for the 1999 patients. We use 2019 as the baseline year (dropped due to use of year FE). The coefficient for 2007 is normalized to 0. Vertical line separates treatment period (2008 on) from pre-treatment period.

To better understand the year-specific change, we regress the outcome variable on year dummies, otherwise using the same specification as in Column (4). Year 2019 is the omitted dummy; the coefficient for 2007 is set to 0. Figure 7 reports the results. Prior to 2008, there is very little change in the yearly proportion of cardiac tests performed by integrated physicians. After 2008, integrated physicians performed an increasingly larger proportion of cardiac tests in M-FFS settings. This timing is consistent with a causal relationship between M-FFS financial incentives and integration.

The M-Adv health systems are integrated throughout our sample period, so there is no movement in these systems of cardiologists from nonintegrated to integrated status. Thus, we can assess the plausibility of integration as a mechanism for test movement but lack a control group that would let us assess movement without the M-FFS financial incentives, and thus cannot conduct a formal DiD assessment of whether the rising Payment Ratio is causally related to integration.

## RESULTS FOR HETEROGENEOUS TREATMENT EFFECTS

In this section, we examine the evidence for heterogeneous treatment effects of financial incentives on NCT location across (1) different types of tests and (2) states with different NCT rates.

### Heterogeneous effects across types of cardiac tests

The HBO/Office Payment Ratio varies across the principal NCTs we study. For example, in 2018, this ratio is 2.57 for SPECT, 2.97 for stress echo, 2.63 for regular echo, and 3.94 for stress ECG.^21^ Thus, the financial incentives to move tests to the HBO location vary with type of test. On the other hand, hospitals assess whether to acquire cardiology practices considering all cardiologist activities together, and a cardiologist who moves to integrated status is likely to move several or all test types at the same time, regardless of the test-specific Payment Ratio. The extent to which test-specific ratios predict movement of specific tests to HBO is therefore unclear theoretically and warrants empirical examination. We therefore assess, in Appendix Tables E.5 and E.6, the extent to which test movement to the HBO location varies by type of test. Figure shows the event study results, which confirm the validity of parallel trends assumption.

We see substantial jumps in the HBO Proportion for all test types, but with smaller magnitude for stress ECG—the simplest, lowest-reimbursing test. The smaller coefficient could reflect the convenience to cardiologists of conducting stress ECG in the Office location more often outweighing the financial gain from moving the test to the HBO location.

### Heterogeneous effects based on NCT rates

We explore in this section some factors that may affect cardiologist movement to integrated practice. We hypothesized that many physicians value autonomy and would prefer to remain independent if a nonintegrated practice can sustain sufficient NCT volumes to make independent practice financially viable. If so, then geographic areas with higher NCT rates might see less integration than areas with lower rates.

We divided states into quartiles based on their NCT rates per 1000 patient-years, averaged over 2005–2007.^22^ We hypothesize that the effects of financial incentives on test location to be stronger in states with lower cardiac testing rates.

**FIGURE 8:**
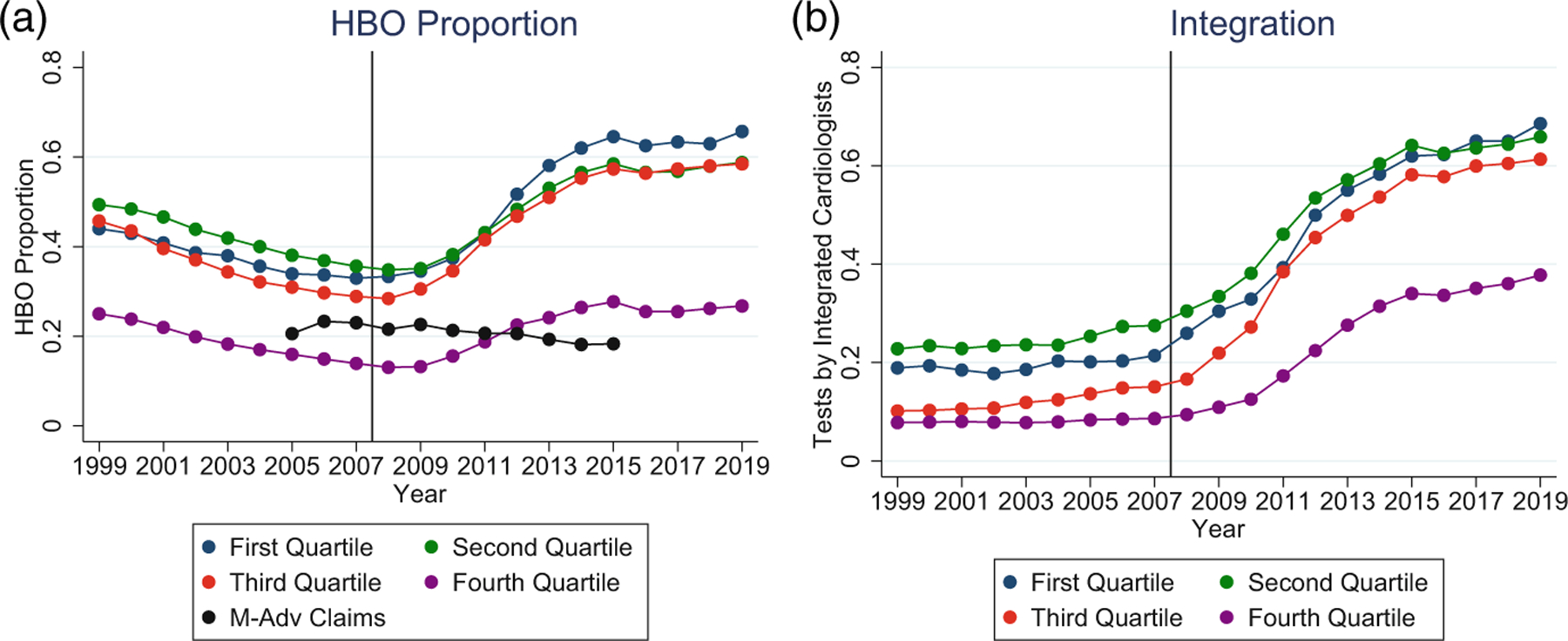
Hospital-based outpatient (HBO) Proportion and integration by testing rate quartile. (a) HBO Proportion for Medicare Fee-for-Service claims over 1999–2019, for states (51, including DC) divided into quartiles based on state-level noninvasive cardiac test (NCT) rates per 1000 patient-years, averaged over 2005–2007. Quartiles are same as in Table 5. For reference, we also plot the HBO Proportion for Medicare Advantage (M-Adv) patients. (b) The proportions of NCTs performed by integrated physicians in each quartile, over 1999–2019.

Figure 8a shows the HBO Proportion over 2005–2015 for states in each testing rate quartile, plus the M-Adv rate for comparison. The states in the first three quartiles have similar HBO Proportions, and similar changes in this proportion over time. In contrast, the fourth quartile (highest testing rates) had a much lower HBO Proportion in 2005 and shows a much smaller change in the proportion over this time period. Figure 8b shows the proportion of NCTs performed by integrated physicians by quartile. The increase over 2008–2016 is much smaller for fourth quartile states. Both panels are consistent with the hypothesis that remaining independent is more financially attractive/viable in states with high NCT rates.

Table 5 shows regression results for each quartile, using a binary treatment-period dummy and full covariates, similar to Table 4, Column (4). In Panel A, the dummy turns on in 2008. In Panel B, we drop 2008–2009, and the treatment-period dummy turns on in 2010. In both panels, the increase in the HBO Proportion is much smaller in quartile 4 than in the other quartiles.^23^

In Appendix Table E.10, we construct quartiles based on testing rates per cardiologist, instead of testing rates per patient. The coefficients on the interaction term are smallest for the quartile with the highest testing rates per cardiologist, which is consistent with the results in Table 5.

## DISCUSSION AND CONCLUSION

We use a DiD design, with M-Adv sites as a control group, to examine the relationship between Medicare payment differentials for the same cardiac tests, performed in HBO versus Office location. We find strong and robust evidence suggesting that the financial incentives created by differential Medicare payments led to significant movement of cardiac tests to the higher-reimbursing HBO setting. We also study the mechanism through which hospitals are able to move cardiac tests to the HBO setting, and provide evidence for acquiring cardiology practices as a principal mechanism.

The acceleration of cardiologists’ vertical integration appears to be an unintended consequence of the CMS decision to reduce facility fees for NCTs performed in Office settings, starting in 2007, without similar changes to fees for HBO tests. CMS saved much less money than it expected to, because many tests migrated to the higher-paying HBO location (Masoudi et al., 2019). The growing payment differential contributed to a substantial change in the industrial organization of cardiology practice. This change appears to be revenue driven.

Our study has important policy implications for M-FFS reimbursement rules. The reductions in Office reimbursement for NCTs led to a large gap in HBO versus Office payments. That gap, to the extent it exceeded the modest differences in HBO versus Office costs, represented economic rents, which hospitals and cardiologists could capture if hospitals employed cardiologists and then moved tests to the HBO location. A large change in the industrial organization of care for cardiac patients was driven by providers’ desire to capture rents.

The Stark laws and physician self-referral laws prohibit hospitals from paying physicians to refer Medicare patients to the hospital for a broad range of medical procedures, including cardiac imaging tests^24^; including paying physicians based on the volume of medical services or drugs that they provide or prescribe.^25^ While these constraints may reduce hospitals’ ability to capture rents by moving test location, our study provides evidence that strong incentives for integration remain. We are unable to observe physician compensation either before or after integration, so cannot study the compensation and other factors that induce cardiologists to become hospital employed.

A natural alternative, which existed for NCTs over 2004–2006, would be “pay parity” for the same procedures—here NCTs—regardless of location. Pay parity for NCTs would give providers incentives to perform these tests in the lower-cost location. It would also remove the current rent-capture incentives for hospital-cardiologist integration. Integration would then be driven, as in other markets, by customary producer incentives to reduce cost and improve quality.

More generally, for visits, tests, and procedures for which Office and HBO treatment are functional substitutes, the CMS approach of reimbursing based on estimated cost in each location, measured separately by different people using different methodologies, can create economic rents due to mis-measurement of actual costs, that providers will then seek to capture. A simple rule—pay the lesser of estimated HBO or Office cost—would avoid the risk of creating rents, reduce Medicare spending, improve allocative efficiency, and provide incentives for integration to occur only when it promises lower cost, better quality, or both.

The Medicare Payment Advisory Commission (MedPac) has recognized since 2012 that higher different reimbursement rates for services performed in the HBO versus Office setting create incentives for healthcare providers to perform services at the higher-paid HBO location and recommended site-neutral payments for E&M services (MedPAC, 2012). In 2018, MedPAC proposed site-neutral payments for all non-acute care (MedPAC, 2018). We estimate extra cost for NCTs at around $600 million in 2017 dollars. For comparison, a recent report from Blue Cross Blue Shield, advocating site-neutral payments estimates extra cost of $3.1 billion annually from diagnostic cardiac catheterization, echo-cardiogram, arthrocentesis, and colonoscopy (BlueCross BlueShield Association, 2023). The Bipartisan Budget Act of 2015 mandated site-neutral payments for certain medical services, starting from 2018. However, the on-the-ground reality has been continued growth in the Payment Ratio for NCTs.

## Supplementary Material

Appendix

## Figures and Tables

**TABLE 1:** Summary statistics for Medicare FFS and Medicare Advantage samples.

	M-FFS (all states)	M-FFS (3 states)	M-Adv	Std. diff.: M-FFS (all states) versus M-Adv	Std. diff.: M-FFS (3 states) versus M-Adv
A: Basic statistics (annual averages)					
Patient years	1,718,346 (38,705)	73,382 (5299)	142,229 (21,439)		
Noninvasive cardiac tests (counts)	369,953 (18,600)	10,357 (219)	13,931 (1808)		
HBO Proportion	0.299 (0.080)	0.432 (0.181)	0.208 (0.018)	1.529	1.739
B: Patient demographics					
Age	76.36 (7.19)	76.343 (7.261)	75.661 (6.945)	0.099	0.096
Comorbidities	2.03 (1.789)	1.774 (1.687)	1.700 (1.709)	0.188	0.044
Female	0.563 (0.496)	0.530 (0.499)	0.505 (0.500)	0.117	0.050
White	0.868 (0.338)	0.939 (0.239)	0.840 (0.367)	0.083	0.321
Black	0.073 (0.260)	0.014 (0.119)	0.030 (0.172)	0.193	−0.108
Hispanic	0.019 (0.138)	0.008 (0.091)	0.044 (0.206)	−0.143	−0.226
Other race	0.036 (0.186)	0.034 (0.182)	0.086 (0.280)	−0.210	−0.217
C: Cardiac test rates (tests per 1000 patient-years, annual averages)					
All cardiac tests	215.5 (13.77)	141.9 (11.62)	101.2 (10.4)	9.40	3.68
Resting echo	130.9 (5.26)	85.0 (4.09)	68.1 (7.76)	9.52	2.72
Any cardiac stress test	84.7 (11.875)	56.9 (10.93)	34.3 (8.777)	4.82	2.28
SPECT	65.0 (10.358)	38.5 (8.69)	17.1 (5.331)	5.82	2.96
Stress ECG	8.87 (1.04)	7.8 (1.36)	14.80 (4.046)	2.01	2.31
Stress echo	10.74 (0.793)	10.6 (1.35)	2.39 (0.608)	11.82	7.84

*Note*: Table reports selected summary statistics for the M-FFS and M-Adv samples. Sample period is 2005–2015. Panels A and C report annual means for the two samples. Panel B reports means for patient demographics over the full sample period, M-Adv data are from Kaiser Permanente Colorado, Kaiser Permanente Northwest, and Group Health. Comorbidities are measured using dummy variables for each of the 17 elements of the Charlson comorbidity index. Standardized difference is difference in means divided by pooled standard deviation (Stata: stddiff.ado). Standard deviations are in parentheses. All standardized differences are statistically significant at the 1% level or better.

Abbreviations: ECG, electrocardiography; HBO, hospital-based outpatient; M-Adv, Medicare Advantage; M-FFS, Medicare Fee-for-Service; SPECT, single photon emission computed tomography.

**TABLE 2:** Continuous difference-in-difference analysis: Effect of Payment Ratio on NCT location.

Panel A: Sample is 50 states and DC							
	Test conducted at HBO location						
Outcome	(1)	(2)	(3)	(4)	(5)	(6)	(7)
M-FFS	0.0947 (0.0939)	**0.254** *** **(0.0723)**	−0.0314 (0.110)	−0.0575 (0.1106)	−0.0253 (0.102)	−0.0561 (0.0998)	−0.0723 (0.104)
Payment Ratio			−0.0335 (0.0227)	NA NA			
M-FFS × Payment Ratio			**0.160** *** **(0.0284)**	**0.173** *** **(0.0287)**			
Payment Ratio (1 lag)					−0.0320 (0.0192)		
M-FFS × Payment Ratio (1 lag)					**0.169** *** **(0.0263)**		
Payment Ratio (2 lags)						−0.0335* (0.0179)	
M-FFS × Payment Ratio (2 lags)						**0.204** *** **(0.0282)**	
Payment Ratio (3 lags)							−0.0368* (0.0195)
M-FFS × Payment Ratio (3 lags)							**0.222** *** **(0.0297)**
Patient demographics	X	X	X	X	X	X	X
Patient comorbidities	X	X	X	X	X	X	X
State fixed effects		X	X	X	X	X	X
Year fixed effects				X			
*R* ^2^	0.010	0.103	0.120	0.130	0.122	0.125	0.126
*N*	3,757,863	3,757,863	3,757,863	3,757,863	3,757,863	3,757,863	3,757,863
Panel B: Sample is OR, WA, and CO (locations of M-Adv sites)							
	Test conducted at HBO location						
Outcome	(1)	(2)	(3)	(4)	(5)	(6)	(7)

M-FFS	0.221 {0.224}	**0.252** ** **{0.026}**	**−0.300** *** **{0.002}**	**−0.341** *** **{0.002}**	**−0.292** *** **{0.002}**	**−0.375** *** **{0.002}**	**−0.409** *** **{0.002}**
Payment Ratio			0.0339 {0.272}	NA NA			
M-FFS × Payment Ratio			**0.327** *** **{0.000}**	**0.333** *** **{0.000}**			
Payment Ratio (1 lag)					−0.033 {0.281}		
M-FFS × Payment Ratio (1 lag)					**0.334** *** **{0.000}**		
Payment Ratio (2 lags)						−0.035 {0.269}	
M-FFS × Payment Ratio (2 lags)						**0.417** *** **{0.000}**	
Payment Ratio (3 lags)							−0.038 {0.244}
M-FFS × Payment Ratio (3 lags)							**0.453** *** **{0.000}**
Patient demographics	X	X	X	X	X	X	X
Patient comorbidities	X	X	X	X	X	X	X
State fixed effects		X	X	X	X	X	X
Year fixed effects				X			
*R* ^2^	0.061	0.101	0.140	0.149	0.147	0.155	0.156
*N*	232,058	232,058	232,058	232,058	232,058	232,058	232,058

*Note*: Regressions use linear probability model, with indicated covariates and state fixed effects (all but Column (1) and year fixed effects (only Column 4) to predict whether test location is in HBO (location dummy = 1) versus Office. Panel A: Sample includes M-FFS claims from 51 states (including DC) and Med-Adv claims from CO, WA, and OR). Standard errors clustered on state in parentheses. Panel B: Sample is limited to CO, WA, and OR. We use the wild-cluster bootstrap and cluster standard errors at state × treatment status level (six clusters). Bootstrapped *p*-values are in curly brackets. Both panels: Sample period is from 2005 to 2015. Payment ratio is measured over 2005–2015 for regressions with no lag of payment ratio, 2004–2014 for regression with 1 lag, etc. Patient demographic covariates are year-of-age dummies, and dummies for gender (male is omitted), Black, Hispanic, other race, and unknown race (White is omitted). Patient comorbidities are dummy variables for the 17 elements of the Charlson comorbidity index. Coefficients on covariates are suppressed. Significant results, at 5% level or better, are in boldface.

Abbreviations: HBO, hospital-based outpatient; M-Adv, Medicare Advantage; M-FFS, Medicare Fee-for-Service; NCT, noninvasive cardiac test.

* *p* < 0.1

** *p* < 0.05

*** *p* < 0.01.

**TABLE 3:** Effects of financial incentives on test location: Binary predictors.

Panel A: Sample is 50 states and DC					
	Test conducted in HBO location				
Outcome	(1)	(2)	(3)	(4)	(5)
Subpanel A.1: Full dataset					
M-FFS	0.0140 (0.0922)	0.00549 (0.0946)	0.00889 (0.0976)	**0.166** ** **(0.0827)**	0.160* (0.0824)
Post-2008 dummy	−0.0193 (0.0238)	−0.0206 (0.0239)	−0.0338 (0.0232)	−0.0287 (0.0235)	NA NA
M-FFS × Post-2008 dummy	**0.101** *** **(0.0274)**	**0.103** *** **(0.0273)**	**0.114** *** **(0.0266)**	**0.115** *** **(0.0273)**	**0.125** *** **(0.027)**
*R*^2^	0.008	0.011	0.016	0.110	0.129
No. of cardiac tests	3,927,605	3,927,605	3,757,863	3,757,863	3,757,863
Subpanel A.2: Excluding 2008 and 2009					
M-FFS	0.0140 (0.0922)	0.00496 (0.0947)	0.00853 (0.0977)	**0.183** ** **(0.0793)**	**0.178** ** **(0.079)**
Post	−0.0240 (0.0237)	−0.0253 (0.0239)	−0.0395 (0.0242)	−0.0329 (0.0247)	NA NA
M-FFS × Post-2010	**0.140** *** **(0.0293)**	**0.142** *** **(0.0293)**	**0.154** *** **(0.0293)**	**0.155** *** **(0.0305)**	**0.160** *** **(0.0302)**
*R*^2^	0.016	0.019	0.024	0.122	0.132
No. of cardiac tests	3,194,614	3,194,614	3,054,206	3,054,206	3,054,206
Patient demographics		X	X	X	X
Patient comorbidities			X	X	X
State fixed effects				X	X
Year fixed effects					X
Panel B: Sample is OR, WA, and CO (locations of M-Adv sites)					
	Test conducted in HBO location				
Outcome	(1)	(2)	(3)	(4)	(5)
Subpanel B.1: Full dataset					
M-FFS	0.060 {0.602}	0.058 {0.700}	0.049 {0.772}	0.084 {0.472}	0.078 {0.526}
Post	−0.021 {0.516}	−0.021 {0.52}	−0.032 {0.43}	−0.028 {0.55}	NA NA
M-FFS × Post	**0.224** *** **{0.000}**	**0.225** *** **{0.000}**	**0.233** *** **{0.000}**	**0.228** *** **{0.000}**	**0.236** **{0.000}**
*R*^2^	0.074	0.076	0.079	0.118	0.134
No. of cardiac tests	261,753	261,753	232,058	232,058	232,057
Subpanel B.2: Excluding 2008 and 2009					
M-FFS	0.060 {0.712}	0.058 {0.658}	0.048 {0.81}	0.083 {0.494}	0.077 {0.532}
Post	−0.026 {0.462}	−0.026 {0.464}	−0.038 {0.246}	−0.032 {0.346}	NA NA
M-FFS × Post	**0.301** *** **{0.000}**	**0.303** *** **{0.000}**	**0.312** *** **{0.000}**	**0.306** *** **{0.000}**	**0.311** **{0.000}**
*R*^2^	0.110	0.112	0.117	0.155	0.164
No. of cardiac tests	216,576	216,576	190,107	190,107	190,112
Patient demographics		X	X	X	X
Patient comorbidities			X	X	X
State fixed effects				X	X
Year fixed effects					X

*Note*: Regressions use linear probability model, with indicated covariates and fixed effects, to predict whether test location is in HBO (location dummy = 1) versus Office. Subpanel A.1: National sample same as Table 2, Panel A. Standard errors clustered on state in parentheses. Subpanel A.2: Same as Subpanel A.1 but exclude 2008–2009 from sample period. Panel B: Sample is limited to CO, WA, and OR. We use the wild-cluster bootstrap and cluster standard errors at state × treatment status level (six clusters). Bootstrapped *p*-values are in curly brackets. Both panels: Significant results, at 5% level or better, are in boldface.

Abbreviations: HBO, hospital-based outpatient; M-Adv, Medicare Advantage; M-FFS, Medicare Fee-for-Service.

* *p* < 0.1

** *p* < 0.05

*** *p* < 0.01

**TABLE 4:** Financial incentives and cardiologist integration.

	Test (in M-FFS) performed by integrated cardiologist			
Outcome	(1)	(2)	(3)	(4)
Panel A: Payment Ratio predictor, full period				
Payment Ratio	**0.218** *** **(0.0200)**	**0.223** *** **(0.0205)**	**0.223** *** **(0.0205)**	**0.223** *** **(0.0203)**
*R*^2^	0.057	0.158	0.158	0.160
No. of cardiac tests	3,314,952	3,314,952	3,314,952	3,191,195
Panel B: Binary predictor, full period				
Post-2008	**0.242** *** **(0.0215)**	**0.248** *** **(0.0220)**	**0.248** *** **(0.0219)**	**0.244** *** **(0.0212)**
*R*2	0.070	0.160	0.160	0.161
No. of cardiac tests	5,911,600	5,911,600	5,911,600	5,521,882
Panel C: Binary predictor, excluding 2008 and 2009				
Post-2010	**0.282** *** **(0.0249)**	**0.290** *** **(0.0254)**	**0.290** *** **(0.0254)**	**0.287** *** **(0.0248)**
*R*^2^	0.095	0.186	0.187	0.188
No. of cardiac tests	5,295,399	5,295,399	5,295,399	4,928,479
State fixed effects		X	X	X
Patient demographics			X	X
Patient comorbidities				X

*Note*: Regressions use linear probability model, with state fixed effects and indicated covariates, to predict whether cardiac test was performed by an integrated cardiologist (integration dummy = 1) versus a nonintegrated cardiologist, regardless of test location. Sample is M-FFS claims for any cardiac test over 1999–2019. Panel A: Uses payment ratio, as primary predictor. Panel B: Uses Post-2008 dummy (=1 for years 2008 and after), as the primary predictor. Panel C: Years 2008–2009 are dropped; uses Post-2010 dummy (=1 for years 2010 and after), as the primary predictor. Standard errors, clustered at the state level, are in parentheses. Significant results, at 5% level or better, are in boldface.

Abbreviation: M-FFS, Medicare Fee-for-Service.

* *p* < 0.1

** *p* < 0.05

*** *p* < 0.01.

**TABLE 5:** Effects of financial incentives on test location (by testing rate quartile).

	Test conducted in HBO location			
Outcome	(1)	(2)	(3)	(4)
Cardiac testing rate quartile	1st	2nd	3rd	4th
Panel A: Full dataset				
M-FFS	0.120 (0.0927)	NA NA	NA NA	NA NA
Post	−0.0295 (0.0241)	−0.0262 (0.0248)	−0.0271 (0.0246)	−0.0277 (0.0250)
M-FFS × Post	0.183*** (0.0384)	0.117*** (0.0296)	0.162*** (0.0299)	0.0800** (0.0282)
*R*^2^	0.119	0.094	0.083	0.040
No. of cardiac tests	426,764	761,650	968,986	1,979,529
Panel B: Excluding 2008 and 2009				
M-FFS	0.132 (0.0909)	NA NA	NA NA	NA NA
Post	−0.0342 (0.0252)	−0.0302 (0.0263)	−0.0307 (0.0262)	−0.0319 (0.0266)
M-FFS × Post	0.238*** (0.0461)	0.159*** (0.0337)	0.218*** (0.0346)	0.109*** (0.0318)
*R*^2^	0.142	0.104	0.106	0.047
No. of cardiac tests	349,263	621,836	786,983	1,608,010
State fixed effects	Y	Y	Y	Y
Patient demographics, comorbidities	Y	Y	Y	Y
Mean HBO Proportion	0.335	0.369	0.298	0.149
Mean NCT rate	150.0	177.5	200.5	247.3
Mean NCT rate per cardiologist	102	138	152	176

*Note*: Regressions and sample are same as Column (4) of Table 4, but treatment group (M-FFS claims) is divided into quartiles based on state NCT rates, averaged over 2005–2007 (13 including CO, OR, WA; 13, 13, and 12 states). Control group (M-Adv from three states) is same for all regressions. All regressions include state fixed effects and indicated covariates (coefficients are suppressed). M-FFS dummy is omitted because collinear with state fixed effects in Columns (2)–(4). Bottom rows indicate mean values for indicated measures, averaged over 2005–2007. Standard errors, clustered at the state level, are in parentheses. Significant results, at 5% level or better, are in boldface.

Abbreviations: HBO, hospital-based outpatient; M-FFS, Medicare Fee-for-Service; NCT, noninvasive cardiac test.

* *p* < 0.1

** *p* < 0.05

*** *p* < 0.01.

## Footnotes

1 In 2010, CMS revised downward its estimates of physician practice expense using a new survey; this contributed to reduced physician reimbursement across many practice areas (Dranove & Ody, 2019). For cardiologists, the 2010 reduction Office payment formed only part of the multi-year reduction in Office payment levels.

2 Whaley et al. (2021) report cross-sectional evidence that integrated physicians do not systemically receive higher income than non-integrated physicians. However, integration could provide physicians with other benefits, including reduced and more predictable work hours, less effort devoted to practice administration, better benefits, and lower financial risk.

3 The Stark Law, or physician self-referral law, prohibits physicians from referring Medicare or Medicaid patients for “designated health services” to any entity with which they have a financial relationship, such as a compensation agreement (42 U.S.C. 1395nn). One designated health service is “radiology and certain other imaging services.”

4 This is a simplification; the HBO location is subject to some regulatory requirements (e.g., ability to maintain negative air pressure in procedure rooms relative to ambient air) that can modestly increase HBO cost.

5 We ended data collection in 3Q 2015 in order to avoid the complications of mapping the transition in 4Q 2015 from the ICD-9CM to the ICD-10CM disease classifications; rates for 2015 are annualized, based on 9 months of data.

6 The Charlson comorbidity measures are: myocardial infarction; congestive heart failure; peripheral vascular disease; cerebrovascular disease; dementia; chronic pulmonary disease; rheumatic disease; peptic ulcer disease; mild liver disease; moderate or severe liver disease; diabetes without chronic complication; diabetes with chronic complication; renal disease; hemiplegia or paraplegia; any malignancy, including lymphoma and leukemia, except malignant neoplasm of skin; metastatic solid tumor; and AIDS/HIV.

7 We did not study coronary computed tomography angiography (CCTA), positron emission tomography (PET), or cardiac magnetic resonance imaging (cardiac MRI) due to low rates during our sample period.

8 Available at: https://www.cms.gov/apps/physician-fee-schedule/search/search-criteria.aspx [accessed April 18, 2022]. PFS is available from 2000; we use Medicare data to estimate payment for 1999.

9 We measure patient enrollment periods, and thus the denominator for testing rates, using monthly data for M-FFS patients (from the Medicare Beneficiary Summary File) and actual eligibility start and end dates for M-Adv patients.

10 As a robustness check, we calculate utilization rates using data pooled across all three sites, thus giving equal weight to each patient, with very similar results. KP CO has roughly twice the patient population of the other two M-Adv sites (see Table 1).

11 Some NCTs are performed by non-cardiologists, principally radiologists. We study only cardiologists.

12 IQVIA advised us that integration data before 2013 are not considered reliable.

13 Other measures exist, but are also unsatisfactory. Forlines (2018) relies on NAMCS surveys. Scott et al. (2017) American Hospital Association annual surveys, which ask whether the hospital employs one or more physicians; this is a binary 0–1 field.

14 We thank the referee for suggesting the specification in Equation (3a).

15 We use the average fraction of each type of tests between 2005 and 2007 as weights in constructing Payment Ratio (and Payment Gap) for all years, so variation in the Payment Ratio comes solely comes from HBO versus Office changes in M-FFS reimbursement amounts. In practice, the proportions of different test types vary over time, with similar changes for HBO and Office testing. See Appendix D.8. The secular changes in test proportions imply changes in the Payment Ratio and thus in the financial incentives to move tests to HBO. See Appendix Figure D.9. However, as this figure shows, the differences between the Payment Ratio, computed using fixed weights, and the ratio using annual weights, are small through 2013, so regression results will be similar using either fixed or annual weights. We confirm this in Appendix Table E.4.

16 An add-on code is a code for a separate payment for a minor part of an underlying procedure. For SPECT during this period, typical billing included the main procedure code and two add-on codes. The main CPT code is 78452 (myocardial perfusion imaging, tomographic [SPECT]), the principal add-on codes are 78478 (myocardial perfusion study with wall motion, qualitative, or quantitative study) and 78480 (myocardial perfusion study with ejection fraction). CMS reimbursed all three codes in the HBO setting at $352 in 2000, $361 in 2001, and $276 in 2002 but the reimbursement for the two add-on codes dropped sharply after 2002. Reimbursement for the add-on codes in the Office setting was much smaller than for the main code throughout this period.

17 Appendix Figure D.1 shows the Office and HBO payment levels separately for each of the four NCTs we study. Appendix Figure D.2 shows the HBO and Office Payment Ratio and Payment Gap by type of test. The test-specific trends are similar to the overall trends documented in Figure 1.

18 Figure D.4A shows the HBO proportion separately for each NCT. For all four tests, the HBO proportions were flat through 2009 and begin to rise in 2010, regular echo rising most sharply. Figure D.5 is similar to Figure 2 but limited to the three states with M-Adv sites, and shows a similar pattern, with a falling HBO Proportion over 1999–2007, and a sharp rise in the HBO Proportion beginning in 2010.

19 We have M-Adv data only for 2005–2015; we assume the HBO Proportion in M-Adv over 2016–2019 would have stayed at its 2015 level. We multiply the number of NCTs observed in our 5% M-FFS sample by 20 to obtain a national estimate.

20 These figures omit year FEs. Event study graphs that include year FEs have slightly larger coefficients in the treatment period, consistent with Tables 2 and 3, but are visually similar.

21 Appendix Figure D.2 shows the test-specific Payment Ratios and Dollar Gaps over 1999–2021.

22 For the first to the fourth quartiles, the 2005–2007 NCT rates are 150.0, 177.5, 200.5, and 247.3, respectively. We think that the testing rates between 2005 and 2007 are unaffected by the financial incentive treatment.

23 Appendix Figure D.8 shows the event study results for the effects of financial incentive in each quartile. The results confirm the validity of parallel trends assumption of DiD analysis.

24 42 U.S.C. § 1395nn. (a)(1)(A) “the physician may not make a referral to the entity for the furnishing of designated health services for which payment otherwise may be made under this subchapter,” and (a)(1)(B) “the entity may not present or cause to be presented a claim under this subchapter or bill to any individual, third party payor, or other entity for designated health services furnished pursuant to a referral prohibited under subparagraph (A).”

25 See, for example, *U.S. ex rel. Baklid-Kunz v. Halifax Hosp. Med. Ctr., No. 6:09-CV-1002-ORL-31, 2012 WL 5415108 (M.D. Fla. Nov. 6, 2012)* (Stark law violation when hospitals paid oncologists incentive bonus based on the value of the prescription drugs and tests that the oncologists ordered); *U.S. ex rel. Drakeford v. Tuomey Healthcare Sys., Inc., 675 F.3d 394 (4th Cir. 2012)* (Stark law violation where a hospital agreed to pay physicians compensation above fair market value in exchange for physicians’ referral of lucrative outpatient procedures).
