# Supplementary material for: Effect of financial incentives on hospital-cardiologist integration and cardiac test location
Source: J Empir Leg Stud. Author manuscript; Available in PMC 2024 Aug 23. (PMC11343569; doi:10.1111/jels.12359)
Supplement: Appendix [file NIHMS1956308-supplement-Appendix.docx]

**Appendix for**

**Effect of Financial Incentives on Hospital-Cardiologist Integration**

**and Cardiac Test Location**

Andy Ye Yuan

Northwestern University, Pritzker School of Law

Ali Moghtaderi

George Washington University, Milken Institute School of Public Health

Timea Viragh

Northwestern University, School of Education and Social Policy

David J. Magid

School of Public Health, University of Colorado

Luo Qian

George Washington University, Milken Institute School of Public Health

Bernard Black

Northwestern University, Pritzker School of Law, Institute for Policy Research, and Kellogg School of Management

(Draft March 2023)

**Northwestern University, Pritzker Law School**

Law and Economics Research Paper No. 22-04

**Northwestern Institute for Policy Research**

Working paper No. 22-xx

*This Appendix can be downloaded without charge from SSRN at:*

<http://ssrn.com/abstract=2858925>

*The article can be downloaded without charge from SSRN at:*

<http://ssrn.com/abstract=2811110>

**Online Appendix**

# Additional Background and Methodological Details

## Non-Invasive Cardiac Tests Studied

We study the following tests:

- transthoracic echocardiography (TTE) and transesophageal echocardiography (TEE) are forms of echocardiography, performed to assess cardiac function and structures. TTE is far more common. We refer to both as resting *echo*, to distinguish them from stress echo.
- Three types of cardiac stress tests:

1. Stress electrocardiography (*stress ECG*). This is the simplest and cheapest cardiac stress test but provides no images of the heart.
2. Stress echocardiography (*stress echo*) combines stress ECG with cardiac imaging using echocardiography.
3. Single photon emission computed tomography (*SPECT*) combines stress ECG with imaging using an injected radioactive isotope.

We refer to all four types of tests together as “Any Cardiac Test.” Stress echo and SPECT, when used with stress ECG, enhance ability to detect or rule our coronary artery disease but are more expensive and require more expertise for interpretation. For many patients, SPECT and stress echo are functional substitutes, and the HBO and Office locations are also functional substitutes.

Cardiac tests can be billed for under multiple CPT codes. In deciding which codes to use, we rely on the consensus judgment of two cardiologists who participated in our overall research project, informed by counts of how often each code is used in our Medicare data.^[[1]](#footnote-1)^ The list of CPT codes we used to identify cardiac tests is reported in Table C.1. For the same procedures and years, our CPT code choices are the same as the American Society of Echocardiography (ASE) Newsletter, which reports payments for specific CPT codes for stress echo, stress ECG, and transthoracic echo; and as Song et al. (2015), who study SPECT and transthoracic echo.^[[2]](#footnote-2)^ We assessed the prevalence of each code in our Medicare data for each year. Table C.2 reports the frequencies with which each main CPT code is reported in selected years (1999, 2006, and 2013).

## Details on Calculations of Payment Levels

We provide here additional details on how we compute Medicare FFS payment levels for cardiac tests. We study the following tests: single photon emission computed tomography (SPECT); stress echocardiography (stress echo), stress electrocardiography (stress ECG)), transesophageal echocardiography (TEE) and transthoracic echocardiography (TTE). We refer to TEE and TTE together as transthoracic echo. We identify tests in our Medicare claims data using Current Procedural Terminology (CPT) codes. Table C.1 lists the CPT codes we used to identify these tests. Table C.2 provides counts for selected years for each main code for each test. As Table C.2 shows, although Medicare also provides cardiac test payment rates for Healthcare Common Procedure Coding System (HCPCS) Level II codes, these are almost never used.

In the text, we rely primarily on the Medicare Physician Fee Schedule (PFS), available from the CMS website, for 2000-2021.^[[3]](#footnote-3)^ This source provides payment amounts separately for professional services in reading the test (using modifier 26), a technical component for performing the test (modifier TC), and both combined. We use the combined series to compute reimbursement levels for Office setting, and use the professional services series to compute reimbursement levels for the professional services component of Hospital Outpatient Department (HBO) testing. As a check on our calculations, we use two alternate sources: (i) the American Society of Echocardiography (ASE) Coding and Payment Newsletter, published annually for 2004-2017 (“ASE Newsletter”), contains reimbursement levels for stress echo, stress ECG and transthoracic echo (but not SPECT);^[[4]](#footnote-4)^ and (ii) actual provider payments, extracted from the Carrier files (for Office tests) and a combination of the Carrier and Outpatient files (for HBO tests) for 1999-2015. All three sources provide consistent estimates, shown below. We also compare our figures to those of Song et al. (2015), who report payment levels for SPECT and transthoracic echo.

CMS sets payment levels through an intricate process that is partly technocratic and partly political (Ferrari et al., 2014). Payment levels change annually for each CPT code; there are usually several common CPT codes for each type of test; and the CPT codes often change, as do the rules for which codes can be billed with which other codes.

## PFS calculations and a Worked-Out Example Using SPECT

We calculated payment levels for each of these CPT codes, separately for office and HBO. We provide a worked-out example here for SPECT.

### A.3.1 Office Reimbursement Calculations

The Physician Fee Schedule (PFS) is based on relative value units (RVUs), which CMS assigns annually based on the resources that it believes are consumed by each service. There are three RVU categories: work, practice expense (PE) and malpractice expense (MP). PE has two components: (i) direct practice expense attributed to each service, to cover supplies, clinical staff, and equipment; and (ii) indirect expense, which varies by specialty and provides an estimate of overhead costs. CMS uses surveys to determine indirect practice expenses. CMS used the AMA Socioeconomic Monitoring Survey. This Survey was discontinued after 1999, but CMS continued to use the 1999 survey results for another decade. In 2010 AMA switched to a new survey; we discuss the implications of that switch below.

Some cardiac tests have both main codes and related “add-on codes,” especially earlier in our sample period. To calculate payment levels, we sum the three types of main code and add-on code RVUs. CMS also announces an annual conversion factor (CF), used to turn RVUs into dollar amounts. Some procedures have two commonly used main codes in a given year. In these cases, we use the simple average of the payments for each main code (plus any related add-on codes).

To compute Office reimbursement levels, we use RVU amounts for the professional and technical components combined.

### A.3.2 An Office Example using SPECT

We calculate payment levels for SPECT main codes 78465 for 1999-2009 and 78452 for 2010-2017, and for add-on codes 78478 and 78480 for 1999-2009.

The formula to compute payment using RVUs from CMS website is

Payment = [Work RVU + PE RVU + MP RVU] * CF

The combined physician and technical RVUs for CPT code 78465 for 2006 are: Work = 1.46, PE = 12.34 and MP = 0.67; the conversion factor is 37.9. So combined physician and technical payment for code 78465 for 2006 is: (1.46+12.34+0.67)*$37.9 = $548.38. We compute dollar amounts for both add-on codes in a similar manner. Then, we sum all three payments.

Table C.3 Panel A provides a worked-out example for Office payments for SPECT for 2006 (with add-on codes) and 2013 (without add-on-codes). Overall payment in 2017$ fell by 41% from 2006 to 2013, from $899.45 to $528.40. This reflected a combination of fewer RVUs (drop from 19.52 to 14.76; 24%), and a 10% nominal (22% real) drop in the conversion factor.

### A.3.2 HBO Reimbursement Calculations

To compute HBO payment levels, we sum payment for the professional component, computed as above, plus dollar amounts for the technical component, which CMS provides annually as Ambulatory Payment Classification (APC) rates.

In the HBO setting, we have to treat separately the technical and professional components of each test. To compute HBO reimbursement levels, we sum (i) payment for the professional component, computed as described above but using RVU amounts solely for the professional component; plus (ii) dollar amounts for the technical component, which CMS provides annually as Ambulatory Payment Classification (APC) rates.

### A.3.3 An HBO Example using SPECT

The professional component is computed as described above. For 2006, for main SPECT CPT code 78465, RVUs for the professional component are: Work = 1.46, PE = 0.52 and MP = 0.05. Thus, the professional component for this main SPECT code is (1.46+0.52+0.05) * [conversion factor = 37.9] = $76.93. We similarly compute the professional component for add-on codes 78478 and 78480, and add them to the payment for the main code to obtain total professional payment. For the technical component we use payment levels reported by CMS using Ambulatory Payment Classification (APC) rates.^[[5]](#footnote-5)^ We then sum these two components.

Table C.3, Panel B, provides a worked-out example for SPECT for 2006 and 2013. Overall HBO payment in 2017$ was $873.73 in 2006 and fell by 9% to $795.71 in 2013.

## ASE Newsletter and a Worked-Out Example Using Stress Echo

The ASE Newsletter provides payment levels by year for 2004-2017 for specific CPT codes for stress echo and transthoracic echo, and for 2004-2014 for stress ECG, as well as other procedures we do not study. The 2015, 2016, and 2018 newsletters do not provide payment levels but the 2017 newsletter includes data for 2016. The codes reported in these newsletters provide a check both on which CPT codes we should use and what the payment levels were. We provide here an example for stress echo, using the ASE Newsletter for 2006 and 2013.

### A.4.1 Office Payment Levels

The ASE Newsletter reports payment levels using national-average RVUs and dollar payments (the same approach we use).^[[6]](#footnote-6)^ It provides a table which lists the principal CPT codes for each procedure, and the national average for the professional component, the technical component, and the “global” payment (the sum of the two components). For stress echo in 2006, the newsletter reports global payment levels for one main code ($151.59 for code 93350) and one add-on code ($108.01 for code 93015). We sum these two figures, to get total stress echo payment $259.60. The method described in §1.2 uses the same codes and produces the same total of $259.60.

For 2013, the ASE Newsletter reports that two main stress echo codes are used – 93350 and 93351. Code 93351 was introduced in 2009, and captures both the prior “main” and “add-on” components. Starting in 2009, code 93350 is also used with a different add-on code, 93017. Table 1 of this newsletter reports total RVUs for professional and technical components combined; Table 2 reports payment levels of $200 and $234, respectively, and that add-on code 93017 is reimbursed at $44. These match exactly our computation using the method described in § 1.2. To obtain total stress echo payment, we average the payment for main code 93351 ($234) and the combined payment for codes 93350 + 93017 ($244); this average is $239. Averaging is a step that the Newsletter does not take; it only provides payment levels for individual codes. ^[[7]](#footnote-7)^

### A.4.2 HBO Payment Levels

The ASE Newsletter also lets us compute national average payment amounts for HBO tests. We calculate HBO payment levels by: (i) using payment for the professional component based on RVUs (see previous subsection); and (ii) using APC levels for the technical component.

For example, to calculate stress echo payment for 2006, we combine payment for the professional component of main code 93350 ($79.58, from Table 1 of ASE Newsletter) and payment for the technical component at the APC rate ($189.01, from Table 2 of the newsletter). CMS does not provide separate technical component payment for add-on code 93015. Thus, HBO payment = $79.58 + $189.01 = $268.59.

For 2013, the professional component of main code 93350 is paid at $70.00, and for the technical component, the APC rate is $390.00; the sum is $460.00. For main code 93351 the respective numbers are $83.00 and $559.00; the sum is $642.00. There is no separate APC payment for add-on code 93017. We average these two numbers: ($460 + $642)/2 = $551.00.

## Medicare Claims Payments and a Worked-Out Example for Transthoracic Echo

As a third approach, we estimate payments using claim-level data from the Carrier and Outpatient files. These files provide information on CMS payments to providers, and on the patient payment (deductible and copayment). We sum the two to get total payment. For 1999, we use this as our primary source, rather than PFS, for 1999 because we were able to find PFS payment levels only from 2000 on. We provide an example below for transthoracic echo.

We refer to TTE and TEE together as transthoracic echo. TTE is much more common than TEE (~400,000 versus ~10,000, see Table C.2), so we compute payment only for TTE. Before 2009, we count payment for the most common main code: 93307. This code is reported with two add-on codes, 93320 and 93325. To compute total payment, we sum payment for these three codes.^[[8]](#footnote-8)^ In 2009 a new “complete” transthoracic echo code is introduced, 93306, which should not be reported with add-on codes. Beginning in 2009 we only calculate payment levels for this combined 93306 code, as the combination of 93307+add-on codes is used only 3-6,000 times depending on year, versus around 400,000 for 93306.

### A.5.1 Office Payments

We compute Office payments using the Carrier file. For each claim line in the Carrier file, we extract: (i) CPT code, (ii) modifiers, to distinguish between technical and professional components, (iii) all payment information (payment to provider, patient deductible, patient co-payment), (iv) location (physician office or hospital outpatient department, identified by location code “11” and “22”, respectively).^[[9]](#footnote-9)^

To calculate the average Office payment for TTE in 2006, we look for all claims involving the relevant CPT codes: 93307 (main code), 93320 (add-on); and 93325 (add-on). Each line can be reported with no modifier, with modifier 26 (for physician component), modifier TC (for technical component), or with both modifiers 26 and TC. A line with no modifier is equivalent to a line with both modifiers. We measure total payment = CMS payment + patient payment (deductible plus copayment). For each CPT code, we average the total payments across all claim lines with either no modifier or both modifiers. For 2006, mean total payments are $207.34 for main code 93307; $92.27 for add-on code 93320 and $134.16 for add-on code 93325. Total payment for TTE in 2006 is the sum of these, or $432.77. For 2013, we use only on CPT code 93306. The average total payment is $190.93.

### A.5.2 HBO Claim-Level Payments

We compute HBO payments using the Carrier file for the physician (professional) component, and the Outpatient file for the technical component. For 2006, mean payments for professional component are $49.27 (for 93307), $20.46 (for 93320) and $4.17 (for 93325); the sum is $73.90. For 2013, the mean professional component payment for code 93306 is $61.92.

For the technical component for 2006, we search for CPT codes 93307, 93320 and 93325 in the Outpatient file. These files do not include modifiers or location codes. We compute average total payment (CMS payment + patient copayment + patient deductible) of $189.01 for code 93307, $99.76 for code 93320, and $89.99 for code 93325; the sum is $378.76. In 2013, only code 93306 is used, with average payment of $390.49. We then sum technical and professional components to determine total HBO payment for each CPT code.

## Stress ECG

Stress ECG is reported without modifiers. It can be reported by either using a global code (93015), or a combination of physician services code (93016 and 93018) and technical code (93017). For Office payments, we use only code 93015 only; the calculation is otherwise similar to § 1.2.

In HBO setting, we calculate the facility fee using the APC value for code 93017, and then add 93016 and 93018 payments (computed as in § 1.2) for the professional component. The table below provides an example for 2006:

| **CPT code** | **Technical Component** | **Professional Component** |
| --- | --- | --- |
| 93017 | 147.78 |  |
| 93016 |  | 24.25 |
| 93018 |  | 15.92 |
| Total | 147.78 | 40.17 |

Total payment = 147.78 + 40.17 = 187.95.

## Comparing payment levels across all sources

Figure C.1 provides Office and HBO reimbursement rates by year, separately for each cardiac test. Separate lines show each available source for each available year. It is visually apparent that all three sources provide consistent estimates.

Our own calculation matches the ASE Newsletter exactly for tests and years where both are available.^[[10]](#footnote-10)^ The only difference is for stress echo, in Office setting, in 2009. This is the first year when code 93351 was used, and the ASE Newsletter reports a slightly higher figure than the one from the CMS website. Actual payments, computed from the Carrier and Outpatient files (M-FFS lines on graphs) also aligns closely with the other sources. Actual payments tend to be slightly higher than our RVU-based estimate in 2000-2003.

## Comparison to Song et al. (2015)

In Figure C.1, we also compare our figures to those reported by Song et al. (2015), who study SPECT and transthoracic echo, using and the same CPT codes that we use. They report 2007-2009, and 2010-2012 averages; we adjust their current dollar values to 2017$. They rely on actual Medicare payments (similar to our third approach). Their data and ours matches closely for Office. For HBO, their values are similar to ours for the professional component but far higher for the technical component. Given that we obtain consistent estimates across three sources for transthoracic echo and two sources for SPECT, it is likely that they went wrong in measuring the technical component for HBO, but we can only speculate

# Identifying Cardiologists

We identify practicing cardiologists using the Individual Healthcare Provider Database developed in a separate project (Moghtaderi, Viragh, and Black, 2019). This database covers essentially all physicians who billed Medicare FFS for services over 1999-2016, and reflects a careful effort to provide physician identifiers, including National Provider Identifier, or NPI, in use since 2007; as well as the predecessor to NPI, known as the Unique Physician Identification Number, or UPIN.

*One major data source* for the Individual Healthcare Provider Database is the National Plan and Provider Enumeration System (NPPES). NPPES provides data on broad “groupings” of healthcare providers, a “classification” within each grouping, and “specialization” within each classification. The relevant grouping for cardiologists is Allopathic & Osteopathic Physicians. The relevant classification is internal medicine, which contains the following specializations related to cardiology:

1. Taxonomy code: 207RA0001X: Advanced Heart Failure and Transplant Cardiology

NPPES definition: Specialists in Advanced Heart Failure and Transplant Cardiology would participate in the inpatient and outpatient management of patients with advanced heart failure across the spectrum from consideration for high-risk cardiac surgery, cardiac transplantation, or mechanical circulatory support, to pre-and post-operative evaluation and management of patients with cardiac transplants and mechanical support devices, and end-of-life care for patients with end-stage heart failure.

1. Taxonomy code: 207RC0000X: Cardiovascular Disease

NPPES definition: An internist who specializes in diseases of the heart and blood vessels and manages complex cardiac conditions such as heart attacks and life-threatening, abnormal heartbeat rhythms.

1. Taxonomy code: 207RC0001X: Clinical Cardiac Electrophysiology

NPPES definition: A field of special interest within the subspecialty of cardiovascular disease, specialty of Internal Medicine, which involves intricate technical procedures to evaluate heart rhythms and determine appropriate treatment for them.

1. Taxonomy code: 207RI0011X: Interventional Cardiology

NPPES definition: An area of medicine within the subspecialty of cardiology, which uses specialized imaging and other diagnostic techniques to evaluate blood flow and pressure in the coronary arteries and chambers of the heart and uses technical procedures and medications to treat abnormalities that impair the function of the cardiovascular system.

There is also a specialty under “Pediatrics”, which is not useful with a Medicare dataset:

1. Taxonomy code: 2080P0202X: Pediatric Cardiology

NPPES definition: Pediatric cardiologist provides comprehensive care to patients with cardiovascular problems. This specialist is skilled in selecting, performing and evaluating the structural and functional assessment of the heart and blood vessels, and the clinical evaluation of cardiovascular disease.

There is also a specialty under "Pediatrics", which is not useful with a Medicare dataset:

1. Taxonomy code: 2080P0202X (8.5% of cardiologist taxonomy codes in NPPES)

Specialty: Pediatric Cardiology

Definition: A pediatric cardiologist provides comprehensive care to patients with cardiovascular problems. This specialist is skilled in selecting, performing and evaluating the structural and functional assessment of the heart and blood vessels, and the clinical evaluation of cardiovascular disease.

We use these six taxonomy codes to identify 39,687 potential cardiologists. We use 2021.08 NPPES, which we “back-fill” with information of deactivated NPIs using all prior years back through 2007. But NPPES is incomplete, because a significant number of physicians do not realize that they should use the NPPES top level grouping for “allopathic and osteopathic physicians,” and instead choose the grouping of “other,” and if they go down this road, they never report a specialty.

*A second source* for identifying cardiologists is Medicare Provider Enrollment, Chain, and Ownership Status (PECOS) public use files, available from 2016 on, and updated quarterly. We use the PECOS base file, which contains information on NPI, Name, Gender, State, specialty, Enrollment id and PAC (Provider Associate Control) id. The PECOS base file allows only one specialty, but additional specialties can be listed in the “Secondary Specialty Subfile,” with enrollment id and additional specialties with no limit on number of specialties. We use both files to identify specialties. We use the following Specialty codes:

1. 14-06: Practitioner - Cardiovascular Disease (Cardiology)
2. 14-21: Practitioner - Cardiac Electrophysiology
3. 14-C3: Practitioner - Interventional Cardiology
4. 14-C7: Advanced heart failure and transplant cardiology

We use the 2021.03 version of PECOS. As we did for NPPES, we generate a backfilled PECOS file, including providers who retire or otherwise drop out of the database in more recent versions. We can identify 36,622 cardiologists from this source.

The third source in identifying cardiologists is Medicare Data on Provider Practice and Specialty (MD-PPAS) that provides annual datasets. MD-PPAS provides a specialty code, but is based on PECOS, so is not a useful source once PECOS becomes available, in 2016. For physicians included in PECOS, MD-PPAS uses the specialty field in PECOS base file (but not the additional specialty subfile). MD-PPAS is useful because it may capture cardiologists who dis-enrolled (e.g., retired) prior to 2016 and were not captured by other sources such as NPPES. As we did with NPPES and PECOS, we create a backfilled MD-PPAS dataset covering all available years (2008-2016). We use the following specialty codes in MD-PPAS:

1. 06: Cardiovascular Disease (Cardiology)
2. 21: Cardiac Electrophysiology
3. C3: Interventional Cardiology

We identified 29,576 cardiologists from MD-PPAS.

He fourth source we use is the ResDAC UPIN (Unique Physician Identification Number) directory, which is available annually for 2003-2007. This source will capture the cardiologists who did not report their specialty correctly in NPPES but in ResDAC UPIN directory. This directory includes a specialty variable (a physician can have up to 5 specialties). We search for “06 - cardiologist.” This directory includes 25,267 distinct UPINs for cardiologists across all years.

The fifth source we use is Medicare Claims Carrier files. Each claim line also includes an indicator for the specialty of the provider that we use. Collectively from all sources, we identified 41,475 cardiologists who billed M-FFS from 1999-2019.

# Additional Tables on Defining Cardiac Tests

**Table C.1: Administrative codes for cardiac imaging procedures**

**Notes:** This table lists the codes we used to identify cardiac imaging procedures.

| **Procedure** | **Code Type** | **Main codes** | **Add-on codes** | **Notes** |
| --- | --- | --- | --- | --- |
| Single-photon emission computed tomography (SPECT) | CPT | 78451, 78452, 78464, 78465, 78468, 78469, G0038, G0039, G0042, G0043 | 78478, 78480 | Codes 78451 and 78452 are used from 2010-2015. Codes 78464, 78465, 78478 and 78480 are used from 1999-2009. G0038, G0039, G0042 and G0043 are used from 1999-2004. All other codes are used from 1999-2015. |
| Stress echocardiogram (Stress Echo) | CPT | 93350, 93351, C8928, C8930, G8961, G8962 | 93015, 93016, 93017, 93018, 93320, 93321, 93325, 93352, A9700, C1759 | Code 93015 is used from 1999-2008. Codes 93351and C8930 are used from 2009-2015. Code C8928 is used from 2008-2015. Codes G8961 and G8962 are used from 2013-2015. All other codes are used from 1999-2015. |
| Stress Electrocardiogram (ECG) | CPT | 93015, 93016, 93017, 93018 | - | All codes are used from 1999-2015. |
| Transesophageal Echo (TEE) | CPT | 93312, 93313, 93314, 93315, 93316, 93317, 93318, C8925, C8926, C8927, G9157 | 93320, 93321, 93325, 93352, A9700, C1579 | Code G9157 is used from 2013-2015. Codes C8925, C8926 and C8927 are used from 2008-2015. Code 93318 is used from 2001-2015. All other codes are used from 1999-2015. |
| Transthoracic Echo (TTE) | CPT | 93303, 93304, 93306, 93307, 93308, C8921, C8922, C8923, C8924, C8929 | - | Codes 93306 and C8929 are used from 2009-2015. All other codes are used from 1999-2015. |

**Table C.2: Procedure counts for cardiac imaging CPT codes, for selected years**

**Notes:** Table shows procedure counts for M-FFS 5% random sample, for main CPT and HCPCS level 2 codes for cardiac imaging tests, for selected years: 1999, 2006, and 2013.

| **SPECT main code counts** | | | | **Stress Echo main code counts** | | | |
| --- | --- | --- | --- | --- | --- | --- | --- |
| **Year** | **1999** | **2006** | **2013** | **Code** | **1999** | **2006** | **2013** |
| **CPT codes** |  |  |  |  |  |  |  |
| 78464 | 6,880 | 4,763 | NA | 93350 | 24,617 | 25,985 | 7,519 |
| 78465 | 97,183 | 183,180 | NA | 93351 | NA | NA | 14,626 |
| 78451 | NA | NA | 2,539 |  |  |  |  |
| 78452 | NA | NA | 124,789 |  |  |  |  |
| 78468 | 259 | 49 | 1 |  |  |  |  |
| 78469 | 288 | 106 | 68 |  |  |  |  |
| **HCPCS level 2 codes** | |  |  |  |  |  |  |
| G0038 | 0 | NA | NA | C8928 | NA | NA | 0 |
| G0039 | 0 | NA | NA | C8930 | NA | NA | 0 |
| G0042 | 1 | NA | NA | G8961 | NA | NA | 0 |
| G0043 | 0 | NA | NA | G8962 | NA | NA | 0 |
| **TEE main code counts** | | | | **TTE main code counts** | | | |
| **Year** | **1999** | **2006** | **2013** | **Code** | **1999** | **2006** | **2013** |
| **CPT Codes** |  |  |  |  |  |  |  |
| 93312 | 8,529 | 12,815 | 15,178 | 93303 | 201 | 431 | 809 |
| 93313 | 939 | 1,506 | 1,502 | 93304 | 42 | 43 | 101 |
| 93314 | 1,024 | 1,132 | 1,372 | 93306 | NA | NA | 401,990 |
| 93315 | 87 | 114 | 103 | 93307 | 275,610 | 421,474 | 3,245 |
| 93316 | 16 | 10 | 14 | 93308 | 3,460 | 6,714 | 11,324 |
| 93317 | 22 | 45 | 49 |  |  |  |  |
| 93318 | NA | 408 | 357 |  |  |  |  |
| **HCPCS level 2 codes** | |  |  |  |  |  |  |
| C8925 | NA | NA | 0 | C8921 | 0 | 0 | 0 |
| C8926 | NA | NA | 0 | C8922 | 0 | 0 | 0 |
| C8927 | NA | NA | 1 | C8923 | 0 | 0 | 0 |
| G9157 | NA | NA | 30 | C8924 | 0 | 0 | 0 |
|  |  |  |  | C8929 | NA | NA | 0 |

**Table C.3: CPT Codes and Payment levels for SPECT**

**Panel A: Office Payment Computation for SPECT, 2006 and 2013**

**Notes:** Examples of payment level computation: SPECT for 2006 (when add-on codes were used), and 2013 (add-on codes no longer used)

| **Year** | **CPT code** | **Work RVU** | **PE RVU** | **MP RVU** | **RVU total** | **Conversion Factor** | **Payment (current $)** | **Payment (2017 $)** |
| --- | --- | --- | --- | --- | --- | --- | --- | --- |
| 2006 | 78465 | 1.46 | 12.34 | 0.67 | 14.47 | 37.8975 | $548.38 | $666.76 |
|  | 78478 (add-on) | 0.62 | 1.79 | 0.12 | 2.53 |  | $95.88 | $116.58 |
|  | 78480 (add-on) | 0.62 | 1.78 | 0.12 | 2.52 |  | $95.50 | $116.12 |
|  | **Total** | **2.70** | **15.91** | **0.91** | **19.52** | **37.8975** | **$739.76** | **$899.45** |
|  |  |  |  |  |  |  |  |  |
| 2013 | 78452 | 1.62 | 13.05 | 0.09 | 14.76 | 34.023 | **$502.18** | **$528.40** |

**Panel B: HBO Payment Computation for SPECT, 2006 and 2013**

| **Year** | **CPT code** | **Work RVU** | **PE RVU** | **MP RVU** | **RVU total** | **Conversion Factor** | **Payment (current $)** | **Payment (2017 $)** |
| --- | --- | --- | --- | --- | --- | --- | --- | --- |
| 2006 | Technical component | | | | | | | |
|  | 78465 |  |  |  |  |  | $397.11 | $482.84 |
|  | 78478 (add-on) |  |  |  |  |  | $89.50 | $108.82 |
|  | 78480 (add-on) |  |  |  |  |  | $89.50 | $108.82 |
|  | **Subtotal TC** |  |  |  |  |  | **$576.11** | **$700.48** |
|  | Professional component | | | | | | | |
|  | 78465 | 1.46 | 0.52 | 0.05 | 2.03 | 37.8975 | $76.93 | $93.54 |
|  | 78478 (add-on) | 0.62 | 0.23 | 0.02 | 0.87 |  | $32.97 | $40.09 |
|  | 78480 (add-on) | 0.62 | 0.22 | 0.02 | 0.86 |  | $32.59 | $39.63 |
|  | **Subtotal Prof** | **2.70** | **0.97** | **0.09** | **3.76** |  | **$142.49** | **$173.25** |
|  | **Total TC + Prof** |  |  |  |  |  | **$718.60** | **$873.73** |
|  |  |  |  |  |  |  |  |  |
| 2013 | Technical component | | | | | | | |
|  | 78452 |  |  |  |  |  | $679.68 | $715.17 |
|  | Professional component | | | | | | | |
|  | 78452 | 1.62 | 0.58 | 0.05 | 2.25 | 34.023 | $76.55 | $80.55 |
|  | **Total TC + Prof** |  |  |  |  |  | **$756.23** | **$795.71** |

# Additional Figures

**Figure D.1: Comparison of Different Sources for M-FFS Payment Levels, by Type of Test**

**Notes:** Payment rates for specific cardiac tests, using data from Physician Fee Schedule Search (PFS), American Society of Echocardiography (ASE), actual M-FFS payments (M-FFS) and Song et al. (2015), where applicable. Rates are in 2017 US dollars. ASE data ends in 2014 for Stress ECG and is missing for 2015 for stress echo and transthoracic echo.

**Figure D.2: HBO and Office Payments by Cardiac Test Type**

**Notes:** The sample period is from 2009 to 2022. The red dashed lines represent the period where we have both Medicare FSS and Medicare Advantage claim data. In Panel A, each data point represents the ratio of Medicare reimbursement payment for cardiac tests conducted at HBO settings and physician offices. In Panel B, each data point represents the dollar gap of Medicare reimbursement for cardiac tests conducted at HBO settings and physician offices.

**Panel A: HBO and Office Payment Ratios**

**Panel B: HBO and Office Payment Dollar Gap**

**Figure D.3:** **The Shares of Tests Performed by Integrated and Non-Integrated Physicians in HBO and Office (By Different Types of Tests)**

**Notes:** The sample include M-FFS claims from 1999 to 2019. Panel (A) shows the type-specific shares of HBO tests performed by integrated cardiologists. Panel (B) shows the type-specific shares of office tests performed by integrated cardiologists.

**Figure D.4: HBO Proportion and Integration by Types of Cardiac Tests**

**Notes:** Figure D.4(A) shows HBO Proportion for M-FFS claims for four different types of Cardiac tests. For reference, we also plot the HBO Proportion for M-Adv patients. The sample period for M-FFS claims is from 1999 to 2019. The sample period for M-Adv claims is from 2005 to 2015. Figure D.4(B) shows the shares of tests performed by integrated physicians by different types of cardiac tests. We are unable to calculate these shares using M-Adv claims data which do not consist of physician identifiers.

**Figure D.5: HBO Proportion for M-FFS versus M-Adv (Three States)**

**Note:** Figure shows HBO proportion for M-FFS over 1999-2019 and M-Adv over 2005-2015 for the three states where we have both M-FFS and M-Adv claims (Colorado, Oregon, and Washington). Each data point represents the share of cardiac tests performed in HBO settings. Dashed vertical lines between 2004-2005 and 2015-2016 indicate the start and end of our sample period. Solid vertical line between 2007-2008 indicates start of the treatment period.

**Figure D.6: Event Study Graph for the Effects of Payment Ratio on Test Location (By Test)**

**Notes:** The sample period is from 2005 to 2015. Each figure shows the effects of financial incentive on test location for each type of cardiac test we examined. We estimated Equation (2) using subsamples of different types of cardiac tests. The omitted year is 2007. Standard errors are clustered at state level.


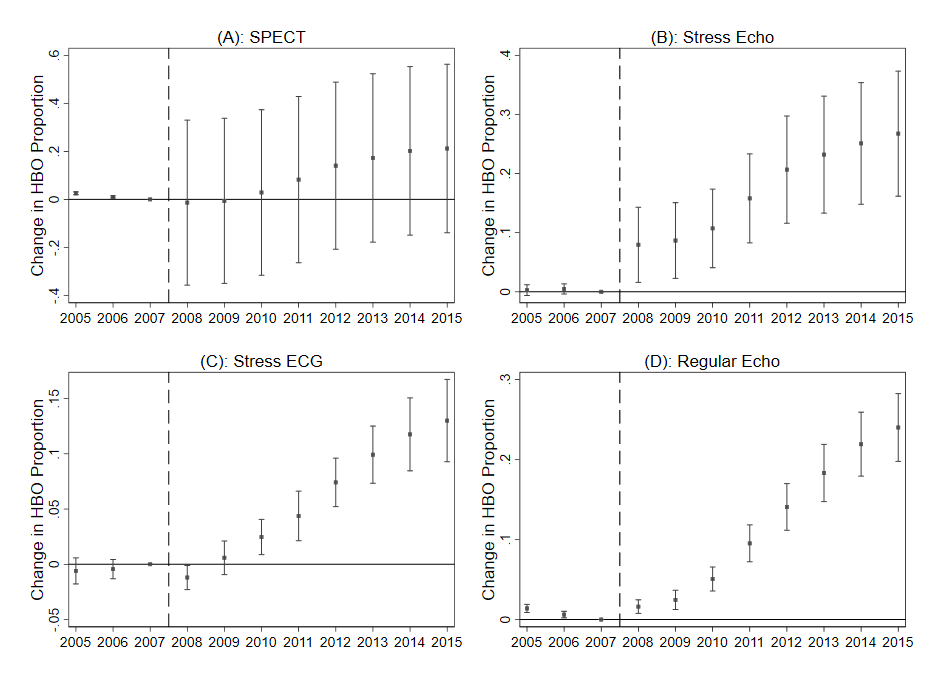


**Figure D.7: Event Study Results for the Effects of Payment Ratio on Test Location (By Pre-2008 State-level Testing Rate)**

**Notes:** The figures present the event study results for the effects of financial incentives on test location among states of different quartiles of pre-2008 testing rates per 1000 patient years. We estimated Equation (2) using subsamples of cardiac tests from states with different quartiles of pre-2008 state-level testing rates. The omitted year is 2007. The standard errors are clustered at state level.


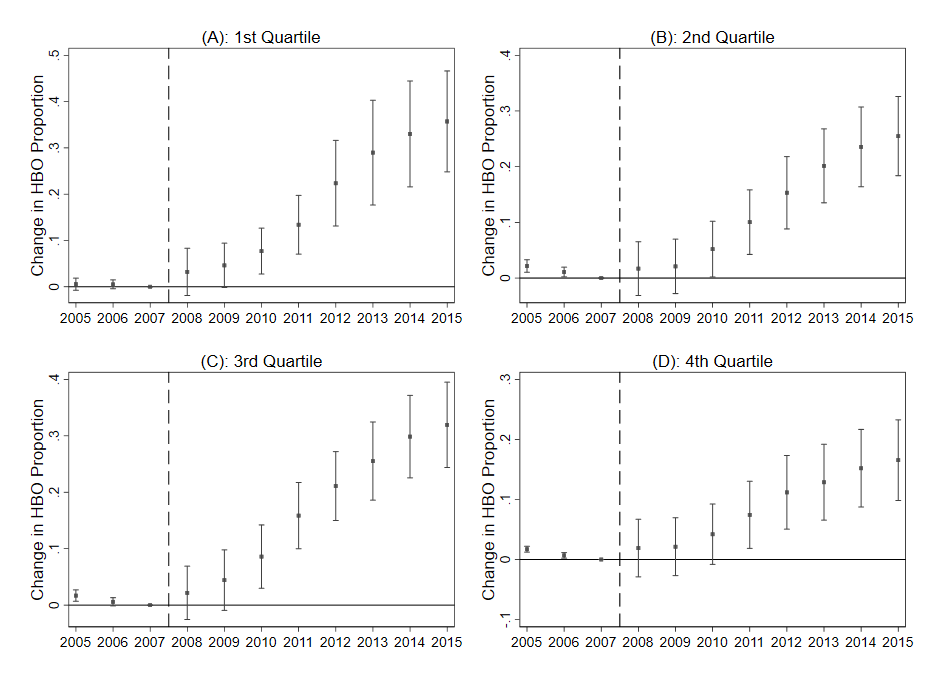


**Figure D.8: Fractions of Each Type of M-FFS Tests at HBO and Office**

**Notes:** The sample period is from 1999 to 2019. The red dashed lines indicate the beginning and the end of the period when we have both M-FFS and M-Adv data. We separately show the yearly fractions of each type of M-FFS tests performed at HBO (left-hand side axis) and Office (right-hand side axis).

**Figure D.9: Payment Ratios using Fixed versus Annually Varying Weights**

Notes: This figure compares annual Payment Ratio and Payment Gap calculated using two different approaches. In the fixed weight approach, we weight the ratio or gap based on the proportion of each type of test averaged over 2005-2007, during the pre-treatment period. In the variable weight approach, we compute the ratio or gap using annual proportions for each type of test.

# Additional Regression Results

## Results Using Alternative Measures of Financial Incentives

**Table E.1: The Effects of *ln*(Payment Ratio) on Test Location**

**Notes**: Regressions use linear probability model, with state fixed effects and indicated covariates, to predict whether test location is in HBO (location dummy =1) versus Office. Regressions are similar to text Table 2, but replace Payment Ratio with *ln*(Payment Ratio) as a predictor variable. Standard errors clustered at state level are in parentheses. Significance levels: *** p<0.01, ** p<0.05, * p<0.1. Significant results, at 5% level or better, in **boldface**.

| **Outcome** | **Test Conducted at HBO Location** | | | | | |
| --- | --- | --- | --- | --- | --- | --- |
|  | (1) | (2) | (3) | (4) | (5) | (6) |
| M-FFS | 0.0947 | **0.254***** | 0.118 | 0.135 | 0.139* | 0.140* |
|  | (0.0939) | **(0.0723)** | (0.0894) | (0.0840) | (0.0813) | (0.0821) |
| Log Payment Ratio |  |  | -0.0547 |  |  |  |
|  |  |  | (0.0384) |  |  |  |
| M-FFS * Log Payment Ratio |  |  | **0.252***** |  |  |  |
|  |  |  | **(0.0471)** |  |  |  |
| Log Payment Ratio (1 Lag) |  |  |  | -0.0507 |  |  |
|  |  |  |  | (0.0316) |  |  |
| M-FFS * Log Payment Ratio (1 Lag) |  |  |  | **0.260***** |  |  |
|  |  |  |  | **(0.0422)** |  |  |
| Log Payment Ratio (2 Lags) |  |  |  |  | -0.0506* |  |
|  |  |  |  |  | (0.0278) |  |
| M-FFS * Log Payment Ratio (2 Lags) |  |  |  |  | **0.302***** |  |
|  |  |  |  |  | **(0.0425)** |  |
| Log Payment Ratio (3 Lags) |  |  |  |  |  | -0.0552* |
|  |  |  |  |  |  | (0.0289) |
| M-FFS *Log Payment Ratio (3 Lags) |  |  |  |  |  | **0.327***** |
|  |  |  |  |  |  | **(0.0435)** |
| State Fixed Effects |  | X | X | X | X | X |
| Patient Demographics | X | X | X | X | X | X |
| Patient Comorbidities | X | X | X | X | X | X |
| R^2^ | 0.010 | 0.103 | 0.118 | 0.121 | 0.125 | 0.126 |
| N | 3757863 | 3757863 | 3757863 | 3757863 | 3757863 | 3757863 |

**Table E.2: Continuous DiD Analysis: Effect of Payment Gap on NCT Location**

**Notes**: Regressions use linear probability model, with state fixed effects and indicated covariates, to predict whether test location is in HBO (location dummy =1) versus Office. Specification and sample are same as text Table 2, Panel A, except principal predictor variable is Payment Gap instead of Payment Ratio. Significance levels: *** p<0.01, ** p<0.05, * p<0.1. Significant results, at 5% level or better, in **boldface.**

| **Outcome** | **Test Conducted at HBO Location** | | | | | |
| --- | --- | --- | --- | --- | --- | --- |
|  | (1) | (2) | (3) | (4) | (5) | (6) |
| M-FFS | 0.0966 | **0.260***** | 0.127 | 0.146* | 0.145* | 0.148* |
|  | (0.0945) | **(0.0734)** | (0.0914) | (0.0846) | (0.0811) | (0.0825) |
| Payment Gap |  |  | -0.0115 |  |  |  |
|  |  |  | (0.00798) |  |  |  |
| M-FFS * Payment Gap |  |  | **0.0496***** |  |  |  |
|  |  |  | **(0.00958)** |  |  |  |
| Payment Gap (1 Lag) |  |  |  | -0.0105 |  |  |
|  |  |  |  | (0.00661) |  |  |
| M-FFS * Payment Gap (1 Lag) |  |  |  | **0.0503***** |  |  |
|  |  |  |  | **(0.00854)** |  |  |
| Payment Gap (2 Lags) |  |  |  |  | -0.0110* |  |
|  |  |  |  |  | (0.00551) |  |
| M-FFS * Payment Gap (2 Lags) |  |  |  |  | **0.0607***** |  |
|  |  |  |  |  | **(0.00851)** |  |
| Payment Gap (3 Lags) |  |  |  |  |  | **-0.0123**** |
|  |  |  |  |  |  | **(0.00525)** |
| M-FFS * Payment Gap (3 Lags) |  |  |  |  |  | **0.0603***** |
|  |  |  |  |  |  | **(0.00770)** |
| State FE |  | X | X | X | X | X |
| Patient Demog. | X | X | X | X | X | X |
| Patient Comorb. | X | X | X | X | X | X |
| R^2^ | 0.010 | 0.103 | 0.116 | 0.118 | 0.121 | 0.120 |
| N | 3757246 | 3757246 | 3757246 | 3757246 | 3757246 | 3757246 |

**Table E.3: The Effects of *ln*(Payment Gap) on Test Location**

**Notes**: Regressions are similar to text Table 2, but replace Payment Ratio with *ln*(Payment Gap) as a predictor variable. Standard errors clustered at state level are in parentheses. Significance levels: *** p<0.01, ** p<0.05, * p<0.1. Significant results, at 5% level or better, in **boldface**.

| **Outcome** | **Test Conducted at HBO Location** | | | | | |
| --- | --- | --- | --- | --- | --- | --- |
|  | (1) | (2) | (3) | (4) | (5) | (6) |
| M-FFS | 0.0966 | **0.260***** | -0.134 | 0.0381 | 0.0618 | 0.0610 |
|  | (0.0945) | **(0.0734)** | (0.140) | (0.118) | (0.0925) | (0.0921) |
| *ln*(Payment Gap) |  |  | -0.0195 |  |  |  |
|  |  |  | (0.0143) |  |  |  |
| M-FFS * *ln*(Payment Gap) |  |  | **0.0735***** |  |  |  |
|  |  |  | **(0.0165)** |  |  |  |
| *ln*(Payment Gap) (1 Lag) |  |  |  | -0.0169 |  |  |
|  |  |  |  | (0.0116) |  |  |
| M-FFS * *ln*(Payment Gap) (1 Lag) |  |  |  | **0.0444***** |  |  |
|  |  |  |  | **(0.0124)** |  |  |
| *ln*(Payment Gap) (2 Lags) |  |  |  |  | -0.00918 |  |
|  |  |  |  |  | (0.00585) |  |
| M-FFS * *ln*(Payment Gap) (2 Lags) |  |  |  |  | **0.0422***** |  |
|  |  |  |  |  | **(0.00739)** |  |
| *ln*(Payment Gap) (3 Lags) |  |  |  |  |  | **-0.00946**** |
|  |  |  |  |  |  | **(0.00452)** |
| M-FFS * *ln*(Payment Gap) (3 Lags) |  |  |  |  |  | **0.0426***** |
|  |  |  |  |  |  | **(0.00606)** |
| State Fixed Effects |  | X | X | X | X | X |
| Patient Demographics | X | X | X | X | X | X |
| Patient Comorbidities | X | X | X | X | X | X |
| R^2^ | 0.010 | 0.103 | 0.113 | 0.111 | 0.114 | 0.114 |
| N | 3757246 | 3757246 | 3757246 | 3757246 | 3757246 | 3757246 |

**Table E.4: The Effects of Payment Ratio (Time-Varying Weights) on Test Location**

**Notes**: Regressions are similar to text Table 2, but Payment Ratio is constructed using annual weights, instead of fixed weights. Standard errors clustered at state level are in parentheses. Significance levels: *** p<0.01, ** p<0.05, * p<0.1. Significant results, at 5% level or better, in **boldface**.

| **Outcome** | **Test Conducted at HBO Location** | | | | | |
| --- | --- | --- | --- | --- | --- | --- |
|  | (1) | (2) | (3) | (4) | (5) | (6) |
| M-FFS | 0.0961 | **0.260***** | -0.0333 | -0.0285 | -0.0540 | -0.0688 |
|  | (0.0943) | **(0.0732)** | (0.112) | (0.103) | (0.101) | (0.105) |
| Payment Ratio |  |  | -0.0369 |  |  |  |
|  |  |  | (0.0236) |  |  |  |
| M-FFS * Payment Ratio |  |  | **0.168***** |  |  |  |
|  |  |  | **(0.0295)** |  |  |  |
| Payment Ratio (1 Lag) |  |  |  | -0.0357* |  |  |
|  |  |  |  | (0.0194) |  |  |
| M-FFS * Payment Ratio (1 Lag) |  |  |  | **0.178***** |  |  |
|  |  |  |  | **(0.0267)** |  |  |
| Payment Ratio (2 Lags) |  |  |  |  | **-0.0370**** |  |
|  |  |  |  |  | **(0.0175)** |  |
| M-FFS * Payment Ratio (2 Lags) |  |  |  |  | **0.209***** |  |
|  |  |  |  |  | **(0.0277)** |  |
| Payment Ratio (3 Lags) |  |  |  |  |  | **-0.0406**** |
|  |  |  |  |  |  | **(0.0191)** |
| M-FFS * Payment Ratio (3 Lags) |  |  |  |  |  | **0.227***** |
|  |  |  |  |  |  | **(0.0291)** |
| State Fixed Effects |  | X | X | X | X | X |
| Patient Demographics | X | X | X | X | X | X |
| Patient Comorbidities | X | X | X | X | X | X |
| R^2^ | 0.010 | 0.103 | 0.120 | 0.123 | 0.126 | 0.126 |
| N | 3769046 | 3769046 | 3769046 | 3769046 | 3769046 | 3769046 |

## Heterogeneous Effects of Financial Incentives

**Table E.5: Continuous DiD Analysis: Effect of Payment Ratio on NCT Location (By Test) Notes**: Regression specification and sample are same as Column (4) of Table 3, Panel B, but the sample is divided into subsamples by cardiac test type. Coefficients on covariates are suppressed. Bottom rows indicate mean M-FFS HBO proportions within indicated years. Standard errors, clustered at the state level, are in parentheses. Significance levels: *** p<0.01, ** p<0.05, * p<0.1. Significant results, at 5% level or better, in **boldface**.

| **Outcome** | **Test Conducted in HBO Location** | | | |
| --- | --- | --- | --- | --- |
|  | (1) | (2) | (3) | (4) |
| Types of Cardiac Tests | Regular Echo | SPECT | Stress Echo | Stress ECG |
| **Panel A: Full Dataset** | | | | |
| M-FFS | 0.0254 | -0.264 | -0.0808 | 0.137 |
|  | (0.0952) | (0.329) | (0.122) | (0.101) |
| Payment Ratio | **-0.0239***** | -0.0409 | **-0.0918**** | 0.0131 |
|  | **(0.00612)** | (0.184) | **(0.0352)** | (0.0142) |
| M-FFS * Payment Ratio | **0.159***** | 0.172 | **0.210***** | **0.0745***** |
|  | **(0.0153)** | (0.186) | **(0.0451)** | **(0.0188)** |
| R^2^ | 0.125 | 0.114 | 0.179 | 0.163 |
| No. of cardiac tests | 2442409 | 1202280 | 194559 | 169460 |
| **Panel B: Excluding 2008 & 2009** | | | | |
| M-FFS | 0.0726 | -0.220 | -0.0280 | 0.152 |
|  | (0.0924) | (0.334) | (0.106) | (0.0994) |
| Payment Ratio | **-0.0215***** | -0.0404 | **-0.0836***** | 0.0112 |
|  | **(0.00540)** | (0.185) | **(0.0293)** | (0.0138) |
| M-FFS * Payment Ratio | **0.151***** | 0.169 | **0.199***** | **0.0739***** |
|  | **(0.0146)** | (0.187) | **(0.0400)** | **(0.0185)** |
| R^2^ | 0.130 | 0.118 | 0.185 | 0.166 |
| No. of cardiac tests | 1992774 | 961844 | 158902 | 139331 |
| State fixed effects | Y | Y | Y | Y |
| Patient demographics, comorbidities | Y | Y | Y | Y |
| 2005-2007 Mean HBO proportion | 0.222 | 0.231 | 0.300 | 0.286 |
| 2010-2015 Mean HBO proportion | 0.341 | 0.349 | 0.395 | 0.349 |

**Table E.6: Effects of Financial Incentives on Test Location: Binary Predictors (By Test)**

**Notes**: Regressions and sample are same as national sample in Column (4) of Table 3, Panel B, but the sample is divided into subsample based on the types of cardiac tests. All regressions include state fixed effects and include indicated covariates (coefficients are suppressed). Bottom rows indicate mean M-FFS HBO proportion, cardiac tests per 1,000 patient years. and spending per beneficiary within each quartile, averaged indicated years. Standard errors, clustered at the state level, are in parentheses). Significance levels: *** p<0.01, ** p<0.05, * p<0.1. Significant results, at 5% level or better, in **boldface**.

| **Outcome** | **Test Conducted in HBO Location** | | | |
| --- | --- | --- | --- | --- |
|  | (1) | (2) | (3) | (4) |
| Types of Cardiac Tests | Regular Echo | SPECT | Stress Echo | Stress ECG |
| **Panel A: Full Dataset** | | | | |
| M-FFS | **0.223**** | -0.0241 | **0.162**** | **0.220**** |
|  | **(0.0883)** | (0.140) | **(0.0716)** | **(0.0849)** |
| Post | **-0.0235***** | 0.00524 | **-0.0873***** | -0.000199 |
|  | **(0.00364)** | (0.176) | **(0.0289)** | (0.00245) |
| M-FFS * Post | **0.116***** | 0.0815 | **0.175***** | **0.0638***** |
|  | **(0.0116)** | (0.178) | **(0.0365)** | **(0.0106)** |
| R^2^ | 0.113 | 0.102 | 0.172 | 0.158 |
| No. of cardiac tests | 2442409 | 1202280 | 194559 | 169460 |
| **Panel B: Excluding 2008 & 2009** | | | | |
| M-FFS | **0.240***** | -0.00469 | **0.178**** | **0.230***** |
|  | **(0.0851)** | (0.137) | **(0.0699)** | **(0.0807)** |
| Post | **-0.0273***** | 0.00125 | **-0.102***** | 0.00408 |
|  | **(0.00508)** | (0.192) | **(0.0375)** | (0.00522) |
| M-FFS * Post | **0.154***** | 0.129 | **0.222***** | **0.0821***** |
|  | **(0.0153)** | (0.194) | **(0.0477)** | **(0.0136)** |
| R^2^ | 0.126 | 0.115 | 0.184 | 0.164 |
| No. of cardiac tests | 1992774 | 961844 | 158902 | 139331 |
| State fixed effects | Y | Y | Y | Y |
| Patient demographics, comorbidities | Y | Y | Y | Y |
| 2005-2007 Mean HBO proportion | 0.222 | 0.231 | 0.300 | 0.286 |
| 2010-2015 Mean HBO proportion | 0.341 | 0.349 | 0.395 | 0.349 |

**Table E.7: The Effects of Payment Ratio on Test Location (By Quartiles of State-Level Pre-2008 Cardiac Test Rates Per Cardiologist)**

**Notes**: Regressions and sample are same as Column (4) of Table 3, but treatment group (M-FFS claims) is divided into quartiles based on state-level testing rates per cardiologist (number of NCTs divided by number of cardiologists), averaged over 2005 to 2007. Quartiles are 13 states incl. OR; 13 incl. CO; 13 incl. WA; and 12 states, respectively. Control group (M-Adv from 3 states) is same for all regressions. All regressions include state fixed effects and indicated covariates (coefficients are suppressed). M-FFS dummy is omitted in col. (4) because it is colinear with state fixed effects. Bottom rows indicate mean HBO proportion, mean NCT rates per 1000 patient year, and mean NCT per cardiologist, averaged over 2005-2007. Standard errors, clustered at the state level, are in parentheses. Significance levels: *** p<0.01, ** p<0.05, * p<0.1. Significant results, at 5% level or better, in **boldface**.

| **Outcome** | **Test Conducted in HBO Location** | | | |
| --- | --- | --- | --- | --- |
|  | (1) | (2) | (3) | (4) |
| Cardiac Testing Rate Quartiles | 1^st^ | 2^nd^ | 3^rd^ | 4^th^ |
| **Panel A: Full Dataset** | | | | |
| M-FFS | **0.333***** | 0.000295 | **0.210***** | NA |
|  | **(0.0252)** | (0.0251) | **(0.0248)** | NA |
| Post | -0.0245 | -0.0248 | -0.0273 | -0.0285 |
|  | (0.0248) | (0.0248) | (0.0248) | (0.0250) |
| M-FFS * Post | **0.139***** | **0.130***** | **0.121***** | **0.100***** |
|  | **(0.0307)** | **(0.0304)** | **(0.0311)** | **(0.0334)** |
| R^2^ | 0.209 | 0.093 | 0.095 | 0.086 |
| No. of cardiac tests | 351222 | 648346 | 1415838 | 1721523 |
| **Panel B: Excluding 2008 & 2009** | | | | |
| M-FFS | **0.339***** | 0.0245 | **0.225***** | NA |
|  | **(0.0253)** | (0.0271) | **(0.0263)** | NA |
| Post | -0.0284 | -0.0284 | -0.0311 | -0.0328 |
|  | (0.0264) | (0.0264) | (0.0263) | (0.0266) |
| M-FFS * Post | **0.175***** | **0.177***** | **0.161***** | **0.137***** |
|  | **(0.0324)** | **(0.0354)** | **(0.0352)** | **(0.0399)** |
| R^2^ | 0.220 | 0.111 | 0.108 | 0.099 |
| No. of cardiac tests | 287322 | 529843 | 1151623 | 1397304 |
| State fixed effects | Y | Y | Y | Y |
| Patient demographics, comorbidities | Y | Y | Y | Y |
| 2005-2007 Mean HBO proportion | 0.335 | 0.369 | 0.298 | 0.149 |
| 2005-2007 Mean NCT rate per 1,000 patient years | 150.0 | 177.5 | 200.5 | 247.3 |
| 2005-2007 Mean NCT rate per cardiologist | 102 | 138 | 152 | 176 |

1. The cardiologist judgments on principal codes were provided by Dr. Steven Farmer, in the course of his work on a prior project involving the M-FFS data, and by Dr. Fred Masoudi. [↑](#footnote-ref-1)
2. The Newsletters are not available from the ASE website. The cardiologist members of our team had saved some earlier newsletters; we obtained others privately from various sources. [↑](#footnote-ref-2)
3. Available at: <https://www.cms.gov/apps/physician-fee-schedule/search/search-criteria.aspx>, accessed 7/13/2018. [↑](#footnote-ref-3)
4. The 2017 Newsletter is available at [http://asecho.org/wordpress/wp-content/uploads/2017/01/2017-Coding-and-Payment-Newsletter-with-Charts_FORMATTED_V3.pdf](http://asecho.org/wordpress/wp-content/uploads/2017/01/2017-Coding-and-Reimbursement-Newsletter-with-Charts_FORMATTED_V3.pdf%20). The 2018 Newsletter (which as no data) is at <http://asecho.org/wordpress/wp-content/uploads/2018/04/2018-ASE-Coding-Newletter-1.pdf>. Earlier versions are not available from the ASE website. The cardiologist members of our team had saved some earlier newsletters; we obtained others privately from various sources. [↑](#footnote-ref-4)
5. CMS publishes an annual table with CPT codes and APC-based payment levels at [https://www.cms.gov/ Medicare/Medicare-Fee-for-Service-Payment/HospitalOutpatientPPS/Hospital-Outpatient-Regulations-and-Notices.html](https://www.cms.gov/%20Medicare/Medicare-Fee-for-Service-Payment/HospitalOutpatientPPS/Hospital-Outpatient-Regulations-and-Notices.html), under “Hospital Outpatient Regulations and Notices.” We use the final Outpatient Prospective Payment System (OPPS) Addendum B tables. These are generally published around November of each year, for the following calendar year. [↑](#footnote-ref-5)
6. This is stated explicitly in the ASE newsletters for some years, and must be true for other years, or we would not achieve the exact matches to ASE payment levels that we in fact achieve. [↑](#footnote-ref-6)
7. The ASE Newsletter reports amounts to the nearest $0.01 in some years, but only to the nearest $1 in other years. [↑](#footnote-ref-7)
8. CMS does not pay separately for the add-on codes under APC for year 2008, so we compute the HBO technical component for 2008 using only main code 93307. [↑](#footnote-ref-8)
9. These location codes are reported by physicians, and do not always match the actual service location. For the purpose for which we use this calculation, as a check on our main calculation using the PFS and APC schedules, we relied on reported location. [↑](#footnote-ref-9)
10. The ASE Newsletters report payment levels in most years in dollars, without cents (2006, used in the example in § 1.3, is an exception). We compute payments to the nearest $.01, so there are minor differences from rounding, which are not visible in the graphs. [↑](#footnote-ref-10)
